# Cytotoxic Assessment of 3,3-Dichloro-β-Lactams Prepared through Microwave-Assisted Benzylic C-H Activation from Benzyl-Tethered Trichloroacetamides Catalyzed by RuCl_2_(PPh_3_)_3_

**DOI:** 10.3390/molecules27185975

**Published:** 2022-09-14

**Authors:** Faïza Diaba, Alexandra G. Sandor, María del Carmen Morán

**Affiliations:** 1 Laboratori de Química Orgánica, Facultat de Farmàcia i Ciències de l’Alimentació, IBUB, Universitat de Barcelona, Avda. Joan XXIII 27-31, 08028 Barcelona, Spain; 2 Departament de Bioquímica i Fisiologia-Seccio de Fisiologia, Facultat de Farmàcia i Ciències de l’Alimentaciò, Universitat de Barcelona, Avda. Joan XXIII 27-31, 08028 Barcelona, Spain; 3 Institut de Nanociència i Nanotecnologia—IN2UB, Universitat de Barcelona, Avda. Diagonal, 645, 08028 Barcelona, Spain

**Keywords:** 3,3-dichloro-β-lactams, cytotoxic activity, squamous cell carcinoma, A431 cell line, hemocompatibility, C-H activation, trichloroacetamides, microwaves, ruthenium catalysts

## Abstract

Natural and synthetic β-lactam derivatives constitute an interesting class of compounds due to their diverse biological activity. Mostly used as antibiotics, they were also found to have antitubercular, anticancer and antidiabetic activities, among others. In this investigation, six new 3,3-dichloro-β-lactams prepared in a previous work were evaluated for their hemolytic and cytotoxic properties. The results showed that the proposed compounds have non-hemolytic properties and exhibited an interesting cytotoxic activity toward squamous cell carcinoma (A431 cell line), which was highly dependent on the structure and concentration of these β-lactams. Among the molecules tested, **2b** was the most cytotoxic, with the lowest IC_50_ values (30–47 µg/mL) and a promising selectivity against the tumor cells compared with non-tumoral cells.

## 1. Introduction

**Figure 1:**
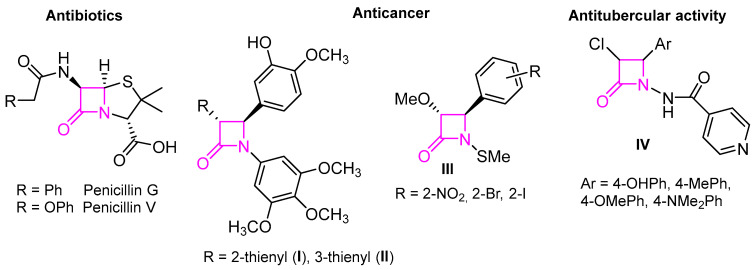
Some β-lactam derivatives with biological activities.

β-Lactam derivatives are the most commonly used bactericidal agents worldwide [1]. Moreover, the β-lactam ring is also found in many other natural and unnatural compounds, with a wide spectrum of activities [2] (Figure 1). For example, in 2011, Meegan et al. reported the synthesis of a series of β-lactams via the Staudinger reaction [3]. Among these, 3-(2-thienyl) **I** and 3-(3-thienyl) **II** displayed the highest potency in human MCF-7 breast cancer cells, with IC_50_ values of 7 nM and 10 nM, respectively, comparable to combretastatin A. Dou et al. have also demonstrated that *N*-thiolated β-lactams **III** have a tumor cell-killing ability through the induction of DNA damage and subsequent apoptosis [4]. Additionally, Hussain et al. reported the synthesis of β-lactam derivatives **IV** with significant antimycobacterial (antitubercular) activity [5]. β-Lactams have been prepared using a wide range of strategies [6,7]. Among these are the Staudinger procedure, also called the Staudinger ketene–imine non-photochemical 2 + 2 cycloaddition [8,9]; the carbonylative ring-opening of aziridines [10]; the Kinugasa reaction, which takes place between terminal alkynes and a nitrone in the presence of copper(I) [11,12,13,14]; and metal-catalyzed C-H insertion of diazoamide carbenes activated by rhodium and ruthenium complexes, among others [15].

**Scheme 1:**
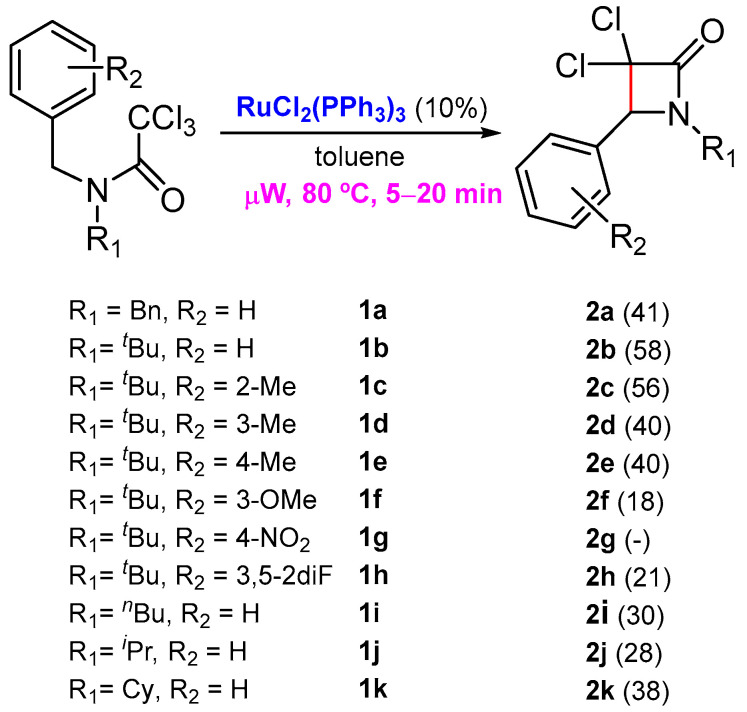
Synthesis of β-lactams through a benzylic C-H activation.

As part of our efforts to prepare new nitrogen-containing heterocycles with cytotoxic activity, a few years ago, we reported an unprecedented ruthenium-catalyzed synthesis of β-lactams from readily available *N*-benzyltrichloroacetamides through benzylic C-H activation [16]. This work was an extension of a previous investigation where we reported the first Grubbs’ second-generation catalyst-promoted intramolecular dearomative ATRC, with trichloroacetamides embodying an electron-rich arene for the preparation of 2-azaspirodecadienes [17,18]. At that time, there was only one example reported by Cramer and co-workers, where a similar saturated C(sp^3^)-C(sp^3^) bond formation was achieved through an asymmetric C-H functionalization from chloroacetamides, catalyzed by palladium(0) [19]. In our investigation, the best results were attained in the presence of a catalytic amount of RuCl_2_(PPh_3_)_3_ under microwave activation, in a very short reaction time (Scheme 1) [16].

Even if the β-lactams prepared using our strategy were isolated with moderate yields, the simplicity of the process and the challenging reaction involved are unprecedented (For NMR spectra of the products involved in this synthetic study see Supplementary Materials). The next step was to study the biological properties of these lactams. In this work, in vitro experiments were performed to determine the biocompatible characterization of six new 3,3-dichloro-β-lactams derivatives **2b**, **2c**, **2e**, **2h**, **2j** and **2k**, regarding their hemolytic and cytotoxic properties in red blood cells (RBCs) and representative skin cell lines, respectively. In view of their putative selective toxicity, both tumoral (A431 cell line) and non-tumoral (HaCaT) cell lines were investigated.

## 2. Results and Discussion

**Figure 2:**
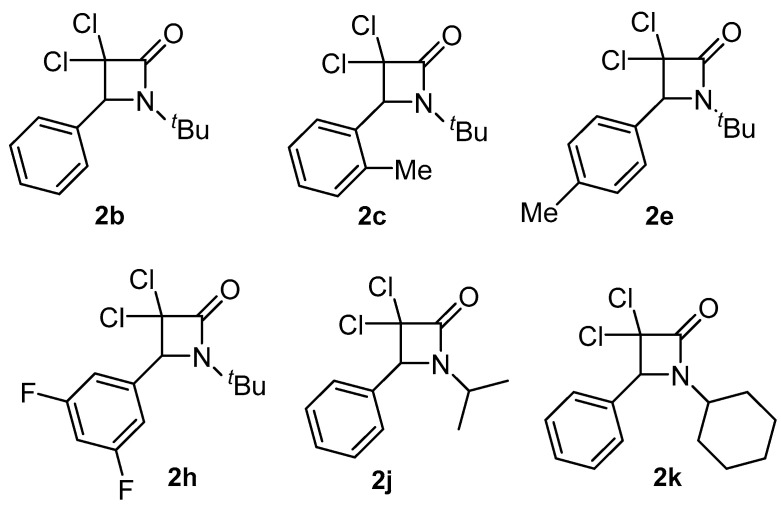
Selected β-lactams for hemocompatibility and cytotoxicity studies.

As was mentioned before with the background reported in the Introduction section, it was of great interest to investigate and characterize some of the β-lactams prepared in our previous investigation, namely **2b**, **2c**, **2e**, **2h**, **2j** and **2k**, for their hemocompatibility and cytotoxic activity (Figure 2).

### 2.1. Hemocompatibility Studies

Following the ISO 10993-4 concerning the biological evaluation of medical devices and their interactions with blood, an in vitro hemocompatibility assay was carried out. Under the assayed conditions, the degree of hemolysis produced by the different compounds by incubation with RBC suspension was determined. The chemical modification of the hemolytic response was evaluated at 2 different concentrations (10 and 80 µg/mL). Table 1 shows representative results as a function of the chemical structure. The degree of hemolysis fluctuated slightly, with values ranging between 0.01 and 0.05% for compounds **2b**–**2j** at concentrations equal to 10 µg/mL. The maximum degree of hemolysis was achieved with compound **2k**, with hemolysis close to 0.25%. By increasing the concentration up to 80 µg/mL, no appreciable increase in the hemolytic response was observed. Only under discrete conditions, hemolysis increases to 2.5% (compound **2b**).

Considering the criteria for which compounds are classified as non-hemolytic (values < 2%), slightly hemolytic (with values 2– 5%) and hemolytic (values >5%), it could be concluded that the proposed compounds showed non-hemolytic properties [20].

### 2.2. Cell Viability-Screening Assays

The interaction between the biological systems and the new materials is of fundamental importance for the study of new materials. After ensuring that the selected β-lactams have a non-hemolytic character, we were very interested in investigating the cytotoxic activity they induced. In vitro cell culture assays are of principal interest in the assessment of the biocompatibility of new compounds. In addition, such initial in vitro analyses provide an excellent way to screen materials before performing in-depth in vivo-based analyses. Among the putative cell lines suitable to perform the present study, commercially available cell lines that include representative cells of the skin were chosen. For this purpose, we used the immortal human keratinocyte (HaCaT) and the squamous cell carcinoma (A431) lines as closely representative skin cell lines with non-tumoral and tumoral characteristics, respectively. The function of the skin includes acting as a barrier, providing protection from physical, chemical and microbiological agents. Keratinocytes represent 95% of the epidermal cells, acting as a structural and barrier function of the epidermis. Their role in skin inflammatory and immunological responses, as well as in wound repair are well-recognized [21]. The spontaneously immortalized human keratinocyte HaCaT cell line from adult skin has been proposed as a model for the study of keratinocyte functions. This work includes another keratinocyte cell line with tumoral characteristics, such as those of squamous cell carcinoma (SCC), namely the A431 cell line, which is important considering that SCC is by far the most common skin cancer and is actually more common than any other form of cancer [22].

**Figure 3:**
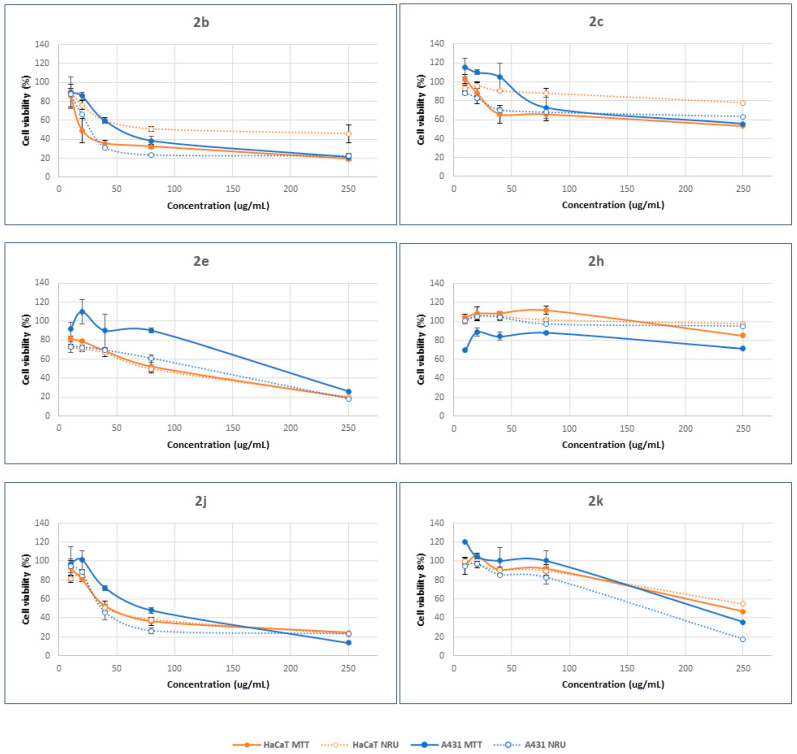
Concentration-dependent viability of HaCaT and A431 cells treated with β-lactam derivatives for 24 h, determined by MTT (solid lines) and NRU (dotted lines) assays. The data correspond to the average of three independent experiments ± standard deviation.

Two different endpoints, 3-(4,5-dimethyl-2-thiazolyl)-2,5-diphenyltetrazolium bromide (MTT) and neutral red uptake (NRU), were used to assess differences in cell-induced cytotoxicity. The former method offers details about the modification of the metabolic activity of mitochondria inside the cells. In the latter case, however, the information derived is related to the interaction with the plasmatic membrane. Dose–response curves were determined by the MTT [23] and NRU [24] assays using HaCaT and A431 cell lines. Cytotoxicity assays were performed at concentrations ranging between 10 and 250 µg/mL. The results demonstrated that the cytotoxic response is highly dependent on the structural characteristics of β-lactam compounds and their concentration. In general, cell viability decreases with increasing concentration, showing a dose–concentration response (Figure 3). However, the final response seems to be a function of the β-lactam derivative.

Compounds **2b**, **2e**, **2j** and **2k** were able to decrease the percentage viability compared to control cells, with values lower than 20%, as a function of cell type and endpoint method. Compounds **2c** and **2h**, however, promoted cell viability by up to 50% in all cases. Moreover, the structure of the β-lactam determined the selectivity against the cell type and the mode of action. Hence, compound **2e** promoted higher cytotoxicity in HaCaT cells than in A431 cells, regardless of the concentration range. Meanwhile, the opposite results were observed in the case of compound **2h**. There were no big differences between the cellular responses when the other β-lactams were considered. As a general trend, these results suggest minor differences between the mode of action of the tested compounds in relation with the plasma membrane and lysosomal accumulation (NRU method) and the modification of the metabolic activity of mitochondria inside the cells (MTT method). In all cases, these compounds can achieve the mitochondrial compartment after alteration of the plasma membrane.

**Figure 4:**
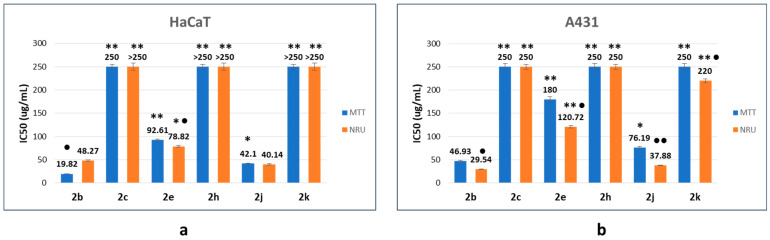
Half-maximal inhibitory concentration (IC_50_) values of the corresponding β-lactams on HaCaT (**a**) and A431 (**b**) cell lines as a function of the endpoint method. The data correspond to the average of three independent experiments ± standard deviation. * *p* < 0.005 and ** *p* < 0.001 indicate significant differences in comparison with compound **2b**. ● *p* < 0.005 and ●● *p* < 0.001 indicate significant differences between endpoint methods for the same compound.

From the fitting of concentration-dependent viability curves, the corresponding half-maximal inhibitory concentration (IC_50_) was determined. The results obtained are summarized in Figure 4 and Table 2. The structural characteristics of β-lactam derivatives seem to be the main factor in the tested compounds’ cytotoxic response. Lactam **2b**, as a reference compound, was the most cytotoxic product, showing the lowest IC_50_ values (20–49 µg/mL and 30–47 µg/mL for HaCaT and A431 cell lines, respectively).

The cellular response depends on the nature of the substituent on the benzene ring and its location. The introduction of two fluorine atoms (compound **2h**) generates a more biocompatible compound, with IC_50_ values close to 250 µg/mL (A431 cell line) or higher than 250 µg/mL (HaCaT cell line), which correspond to the highest tested concentration, in a manner independent of the endpoint method. The location of the methyl group in the benzene ring is also an important parameter concerning cell response. Thus, when the methyl group is in the ortho position (compound **2c**), IC_50_ values are close to 250 µg/mL (HaCaT and A431, MTT endpoint) and higher than 250 µg/mL (HaCaT and A431, NRU endpoint). However, when the methyl substituent is in the para position (compound **2e**), the compound preserves the cytotoxic activity, with IC_50_ values between 78 and 93 µg/mL (HaCaT), and between 120 and 180 µg/mL (A431). Switching from *t*-Bu to *i*-Pr (compound **2j**) has a poor influence on the cytotoxic response, since IC_50_ values between 40 and 42 µg/mL (HaCaT), and between 38 and 76 µg/mL (A431) were found. However, in the case of the cyclohexyl derivative **2k**, limited interaction with the proposed cells was observed, as is indicated by the IC_50_ values close to 220 µg/mL (HaCaT, NRU) or higher than 250 µg/mL (HaCaT, MTT and A431 in all cases).

When the IC_50_ values as a function of the endpoint method were compared, the results obtained suggested that, in general, the interaction with the cell membrane is favored. In almost all cases, IC_50_ values corresponding to the NRU method were lower than those obtained with the MTT endpoint. Moreover, based on the values displayed in Table 2, the selectivity index (SI) seems to depend on the endpoint method. In the case of the MTT method, poor selectivity was detected. In all cases, SI values are equal to or lower than 1, demonstrating no selectivity in the mode of action. However, some selectivity among the proposed cell lines was observed when the NRU endpoint was considered, demonstrating discrete selectivity in the mode of action of almost all compounds (except for compound **2e**).

Such initial in vitro analyses provide an excellent way to screen materials before performing in-depth in vivo-based analyses. In addition, preclinical models such as human cell line model systems may be particularly useful to help predict anticancer drug response and to further improve our understanding of the mechanisms of drug action in cases where there is limited access to clinical samples and/or the cost of obtaining clinical samples to study the drug response is too high. Based on the results obtained, we can confirm that the synthesized β-lactams represent new compounds with antitumoral properties which can be modulated as a function of the synthesized β-lactams’ structure.

## 3. Materials and Methods for the Biological Assays

### 3.1. Materials

Dulbecco’s modified Eagle’s medium (DMEM), fetal bovine serum (FBS), L-glutamine solution (200 mM), trypsin–EDTA solution (170,000 U/L trypsin and 0.2 g/L EDTA), penicillin–streptomycin solution (10,000 U/mL penicillin and 10 mg/mL streptomycin) and phosphate-buffered saline (PBS) were obtained from Lonza (Verviers, Belgium). 3-(4,5-Dimethyl-2-thiazolyl)-2,5-diphenyltetrazolium bromide (MTT) and neutral red dye (NR) were from Sigma–Aldrich (St. Louis, MO, USA). The 75 cm^2^ flasks and 96-well plates were obtained from TPP (Trasadingen, Switzerland). All other reagents were of analytical grade.

### 3.2. Methods

#### 3.2.1. In Vitro Assay with Human Erythrocytes

##### Acquisition and Extraction of the Erythrocytes

Human blood samples were obtained from the Banc de Sang i Teixits de Barcelona (Spain) from the Catalan Department of Health. Blood was deposited in tubes with the anticoagulant EDTA-K3. Blood samples were centrifuged at 3000 rpm at 4 °C for 10 min (Megafuge 2.0 R. Heraeus Instruments, Hanau, Germany) to induce sedimentation. Plasma was extracted with a Pasteur pipette. Next, the residual pellet was washed with PBS at pH 7.4. This procedure was repeated three times to remove residual leukocytes and platelets and to concentrate the erythrocytes. Following the last wash, the erythrocyte suspension was diluted (1:1) in PBS at pH 7.4 to obtain a suitable erythrocyte suspension (cell density of 8 × 10^9^ cell/mL).

##### Hemolysis Assay

The hemolysis assay determined the capability of the different compounds to induce hemolysis of the erythrocyte membrane. Stock solutions of each compound at 1 mg/mL in PBS at pH 7.4 were prepared. Different volumes (10–80 µL) were placed in polystyrene tubes, and an aliquot of 25 µL of the erythrocyte suspensions was added to each tube. The final volume was 1 mL. The tubes were incubated at room temperature under rotatory conditions. Next, the tubes were centrifuged at 10,000 rpm for 5 min. The supernatants’ absorbance at 540 nm (Shimadzu UV-160A, Shimadzu, Duisburg, Germany) was compared with that of the control samples hemolyzed with distilled water (positive control). The negative control was obtained by incubating an aliquot of 25 µL of the erythrocyte suspension with PBS at pH 7.4.

The degree of hemolysis was determined with the following equation:

Hemolysis (%) = 100 × (Abs − Ab_0_)/(Abs_100_ − Abs_0_)

where Abs, Abs_0_ and Abs_100_ are the absorbance of test samples, of the suspension treated with isotonic physiological buffer saline (PBS) and of the suspension of complete hemolysis treated with distilled water, respectively.

### 3.3. Cell Cultures

The immortal human keratinocyte (HaCaT) and the squamous cell carcinoma (A431) cell lines were obtained from Celltec UB. Cells were grown in DMEM medium (4.5 g/L glucose) supplemented with 10% (*v/v*) FBS, 2 mM L-glutamine, 100 U/mL penicillin and 100 µg/mL streptomycin at 37 °C, 5% CO_2_. Cells were routinely cultured in 75 cm^2^ culture flasks and were trypsinized using trypsin–EDTA when the cells reached approximately 80% confluence. The trypan blue assay, which allows a direct identification and enumeration of live (unstained) and dead (blue) cells in a given population, was used to evaluate the viability of the cells in the cell suspension obtained.

### 3.4. Cell Viability-Screening Assays

HaCaT cells (1 × 10^5^ cells/mL) and A431 cells (5 × 10^4^ cells/mL) were grown at the defined densities in the 60 central wells of a 96-well plate. Cells were incubated for 24 h in 5% CO_2_ at 37 °C. Then, the spent medium was removed, and cells were incubated for 24 h with the corresponding compound solutions (1 mg/mL) previously diluted in the minimum amount of DMF (dimethylformamide) and then in DMEM medium supplemented with 5% FBS (100 µL) at the required concentration range (10–250 µg/mL). The viability of the cells upon incubation with the β-lactam derivatives was assayed using 2 different endpoints: NRU and MTT.

#### 3.4.1. NRU Assay

The neutral red uptake (NRU) assay is based on the accumulation of the dye in the lysosomes of viable cells. After the cells were incubated for 24 h with the corresponding systems, the medium was removed, and the solutions were incubated with the NR dye (Sigma-Aldrich, St. Louis, MO, USA) solution (50 µg/mL) dissolved in the medium without FBS and without phenol red (Lonza, Verviers, Belgium) for 3 h. Cells were then washed with sterile PBS, followed by the addition of 100 µL of a solution containing 50% absolute ethanol and 1% acetic acid in distilled water to extract the dye. To promote total dissolution of the dye, plates were placed in a microtiter-plate shaker for 5 min at room temperature. The absorbance of the resulting solutions was measured at 550 nm (Bio-Rad 550 microplate reader, Bio-Rad California, Hercules, CA, USA). Finally, the effect of each treatment was calculated as the percentage of dye uptake by viable cells relative to the control cells (cells without any treatment).

#### 3.4.2. MTT Assay

Only living cells can reduce the yellow tetrazolium salt 3-(4,5-dimethyl-2-thiazolyl)-2,5-diphenyltetrazolium bromide (MTT) to insoluble purple formazan crystals. After 24 h of incubation of the cells with the corresponding NPs, the medium was removed, and 100 µL of MTT (Sigma-Aldrich, St. Louis, United States) in PBS (5 mg/mL) diluted 1:10 in culture medium without phenol red and without FBS (Lonza, Verviers, Belgium) was added to the cells. After 3 h of incubation, the medium was removed. Thereafter, 100 µL of DMSO (Sigma-Aldrich, St. Louis, MO, USA) was added to each well to dissolve the purple formazan crystals. Agitation and determination of the absorbance of the extracted solution were performed under the same conditions, as described in Section 3.4.1. Finally, the effect of each treatment was calculated as the percentage of tetrazolium salt reduction by viable cells relative to the control cells (cells without any treatment).

#### 3.4.3. Selectivity towards Cancer Cells

The corresponding half-maximal inhibitory concentration (IC_50_) values for the different formulations as a function of cell line and endpoint were determined from the fitting of concentration-dependent viability curves.

The corresponding selectivity indexes toward tumoral cells were calculated as the following ratio:

SI = IC_50_ (non-tumoral cell line)/IC_50_ (tumoral cell line)

where HaCaT keratinocytes were used as close representatives of a skin model cell line under non-tumoral conditions.

### 3.5. Statistical Analyses

Experiments were performed three times on independent occasions unless otherwise stated. The results are expressed as means ± standard deviation. One-way analysis of variance (ANOVA) was used to determine statistical differences between data sets, followed by Scheffé post hoc tests for multiple comparisons. IBM SPSS Statistics software version 27.0 (New York, NY, USA) was used to execute statistical analyses. Differences were considered statistically significant at *p* < 0.005 and 0.001. Significant differences were illustrated in the figures with an asterisk or other superscript symbols.

## 4. Conclusions

In summary, 3,3-dichloro-β-lactams **2b**, **2c**, **2e**, **2h**, **2j** and **2k**, prepared through an unprecedented RuCl_2_(PPh_3_)_3_-catalyzed benzylic C-H activation from benzyl-tethered trichloroacetamides, were evaluated for their hemolytic and cytotoxic properties. The results showed that the proposed compounds have non-hemolytic properties and that the cytotoxic response is highly dependent on the structure and concentration of these β-lactams. Compound **2b** was found to be the most cytotoxic of the list, showing the lowest IC_50_ values (20–49 µg/mL and 30–47 µg/mL for HaCaT and A431 cell lines, respectively), with moderate selective toxicity against tumoral cells.

## Figures and Tables

**Table 1:** Percentage of hemolysis induced by compounds **2b**–**2k** as a function of concentration.

Compound	Hemolysis 10 µg/mL	80 µg/mL
**2b**	0.01 ± 0.01	2.5 ± 0.97
**2c**	0.01 ± 0.01	0.01 ± 0.01
**2e**	0.01 ± 0.01	0.2 ± 0.23
**2h**	0.05 ± 0.05	0.12 ± 0.09
**2j**	0.01 ± 0.01	0.02 ± 0.02
**2k**	0.25 ± 0.03	0.30 ± 0.21

**Table 2:** Cell viability assessment: IC_50_ values of the corresponding β-lactams as a function of cell line and endpoint method. Selectivity index against the tumor cell line (A431) in comparison with the non-tumor cell line (HaCaT).

Compound	IC_50_ HaCaT (µg/mL)		IC_50_ A431 (µg/mL)		SI	
	MTT	NRU	MTT	NRU	MTT	NRU
**2b**	19.82	48.27	46.93	29.54	0.42	1.63
**2c**	250	>250	250	250	1.0	>1.0
**2e**	92.61	78.82	180	120.72	0.51	0.65
**2h**	>250	>250	250	250	>1.0	>1.0
**2j**	42.10	40.14	76.19	37.88	0.55	1.06
**2k**	>250	>250	250	220	>1.0	>1.13

## Data Availability

All data are available within the manuscript.

## Supplementary Materials

The following supporting information can be downloaded at https://www.mdpi.com/article/10.3390/molecules27185975/s1, Figure S1: ^1^H and ^13^C spectra of **1e**, Figure S2: ^1^H and ^13^C spectra of **1f**, Figure S3: ^1^H and ^13^C spectra of **1g**, Figure S4: ^1^H and ^13^C spectra of **2a**, Figure S5: ^1^H and ^13^C spectra of **2b**, Figure S6: ^1^H and ^13^C spectra of **2c**, Figure S7: ^1^H and ^13^C spectra of **2d**, Figure S8: ^1^H and ^13^C spectra of **2e**, Figure S9: ^1^H and ^13^C spectra of **2f**, Figure S10: ^1^H and ^13^C spectra of **2h**, Figure S11: ^1^H and ^13^C spectra of **2i**, Figure S12: ^1^H and ^13^C spectra of **2j**, Figure S13: ^1^H and ^13^C spectra of **2k**.
