# Supplementary material for: Cytotoxic Assessment of 3,3-Dichloro-β-Lactams Prepared through Microwave-Assisted Benzylic C-H Activation from Benzyl-Tethered Trichloroacetamides Catalyzed by RuCl2(PPh3)3
Source: Molecules. 2022 Sep 14;27(18):5975. doi: 10.3390/molecules27185975 (PMC9500805; doi:10.3390/molecules27185975)
Supplement: Supplementary file 1 [file molecules-27-05975-s001.zip › molecules-1871348-supplementary.pdf]

Supporting information

for

Cytotoxic Assessment of 3,3-Dichloro-β-Lactams Prepared through Microwave-Assisted Benzylic C-H Activation from Benzyl-Tethered Trichloroacetamides Catalyzed by $RuCl_2(PPh_3)_3$

Faïza Diaba[1,*], Alexandra G. Sandor[1] and María del Carmen Morań[2,3]

faiza.diaba@ub.edu

[1] *Laboratori de Química Orgánica, Facultat de Farmàcia i Ciències de l'Alimentació, IBUB, Universitat de Barcelona, Av. Joan XXIII 27-31, 08028 Barcelona, Spain*

[2] *Departament de Bioquímica i Fisiologia-Seccio de Fisiologia, Facultat de Farmàcia i Ciències de l'Alimentaciò, Universitat de Barcelona, Avda. Joan XXIII 27-31, 08028 Barcelona, Spain*

[3] *Institut de Nanociència i Nanotecnologia – IN2UB, Universitat de Barcelona, Av. Diagonal, 645, 08028 Barcelona, Spain*

- Copies of ^{1}H NMR and ^{13}C NMR spectra of compounds **1**-**2** S2-S14

VNMRS400A_09042018_ZAS088-16-H1
VNMRS400F / Num.Inv. 205984
cdcl3 / Temp: 25C / N.Reg: XXXXXXXXXX
Usuari: san / Mostra: ZAS088-16
Nom: FAIZA DIABA
Data: 09/04/18 / Ope.: F.DIABA

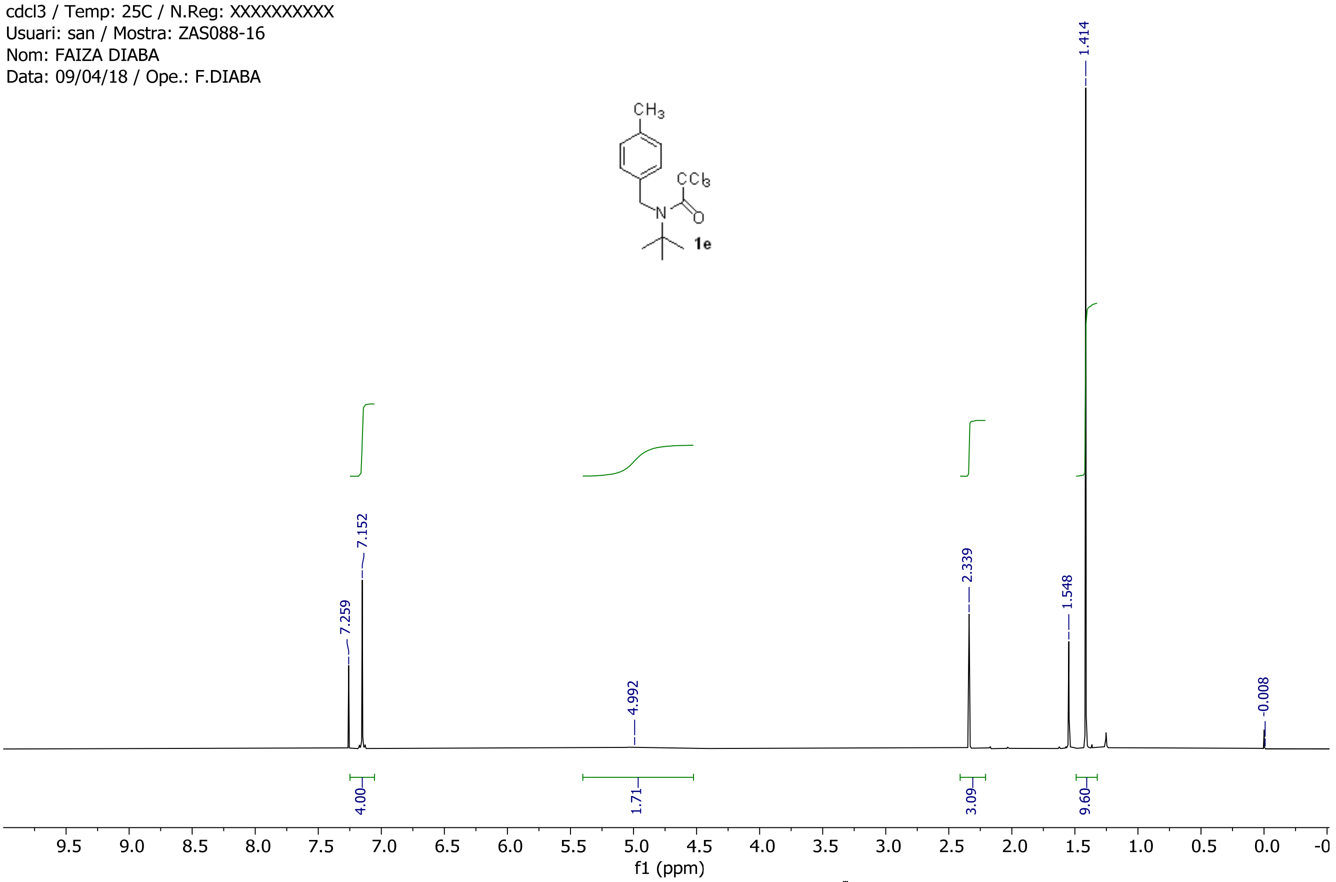

VNMRS400A_10042018_ZAS088-16-C13
VNMRS400F / Num.Inv. 205984
cdcl3 / Temp: 25C / N.Reg: XXXXXXXXXX
Usuari: san / Mostra: ZAS088-16
Nom: FAIZA DIABA
Data: 10/04/18 / Ope.: F.DIABA

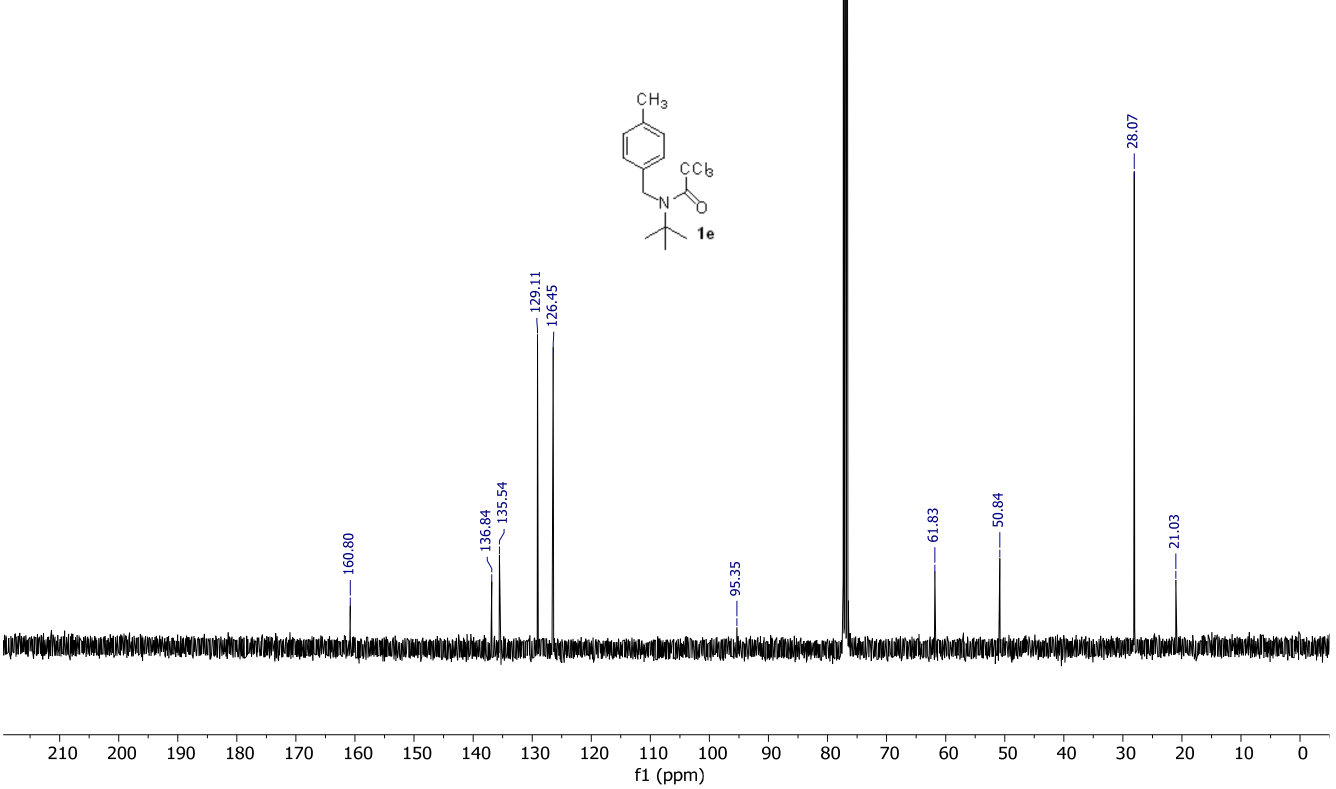

Figure S1

V400A_02022013_XJA103-10-H1
H1 / Mercury-400F
cdcl3 / Temp: 25C / N.Reg: XXXXXXXXXX
Usuari: san / Mostra: XJA103-10
Nom: FAIZA DIABA
Data: 01/02/13 / Ope.: F.DIABA
Experiment: s2pul

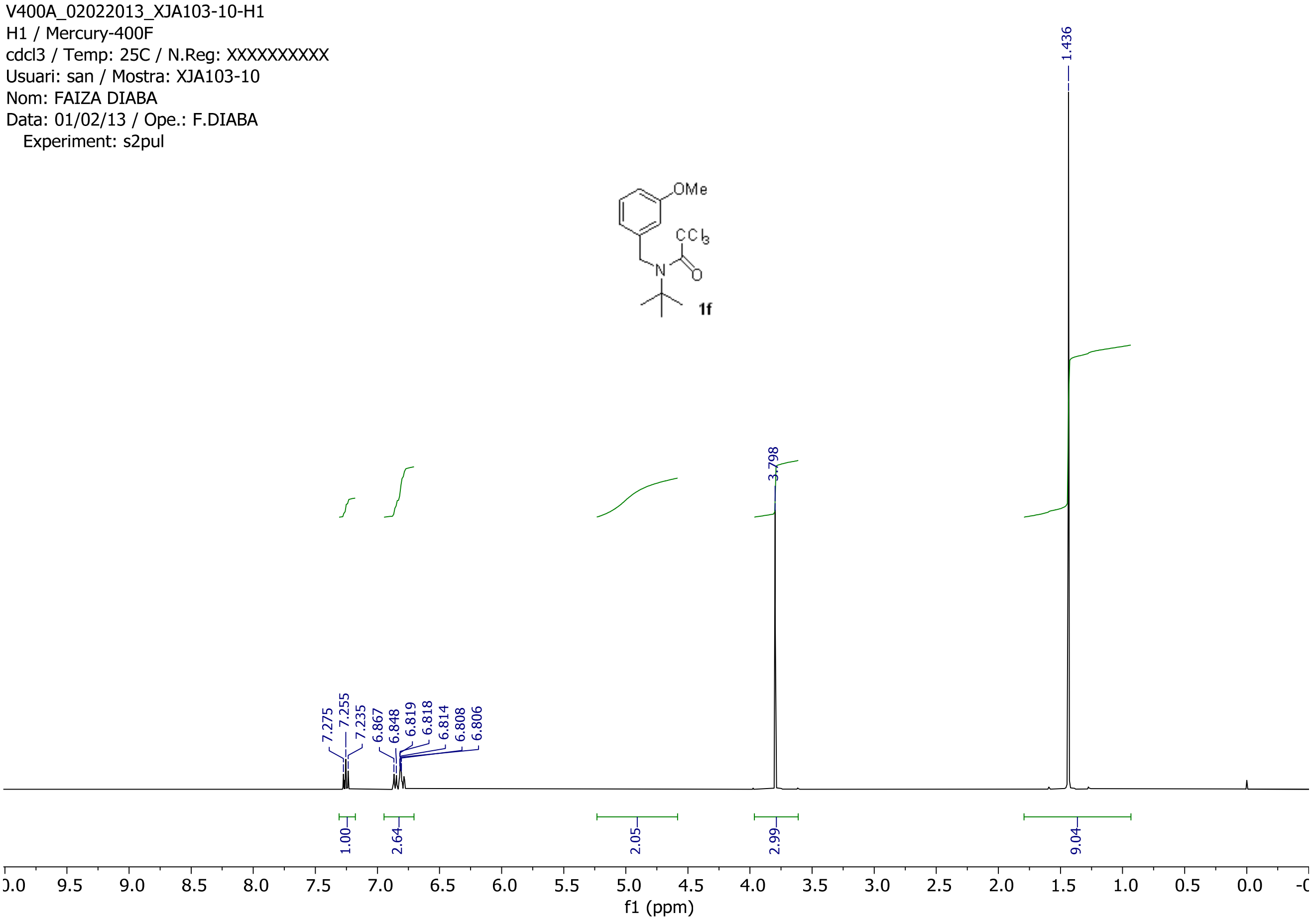

V400A_02022013_XJA103-10-C13
H1 / Mercury-400F
cdcl3 / Temp: 25C / N.Reg: XXXXXXXXXX
Usuari: san / Mostra: XJA103-10
Nom: FAIZA DIABA
Data: 01/02/13 / Ope.: F.DIABA
Experiment: s2pul

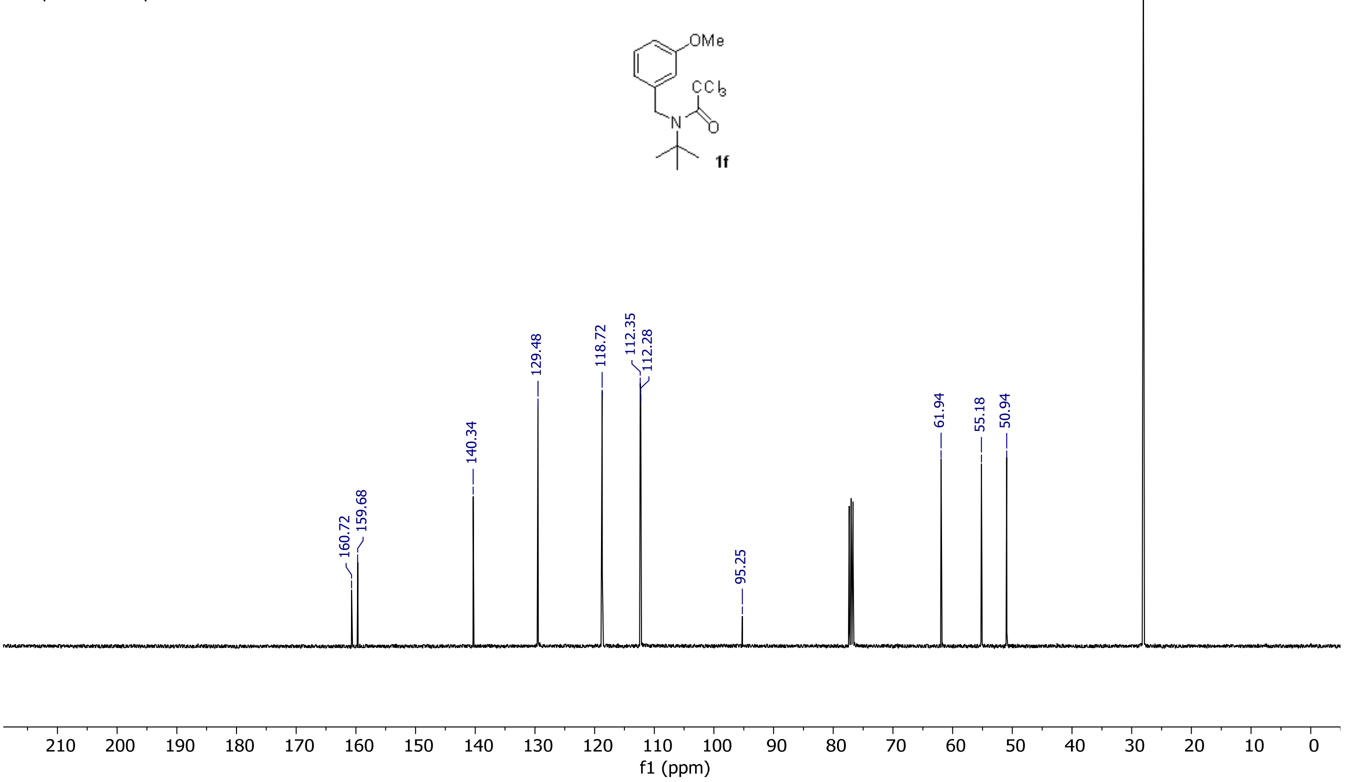

Figure S2

M400AFF_11042018_AS084-2-6-H1
M400F / Num.Inv. 1009191
cdcl3 / Temp: 25C / N.Reg: XXXXXXXXXX
Usuari: san / Mostra: AS084-2-6
Nom: FAIZA DIABA
Data: 11/04/18 / Ope.: F.DIABA

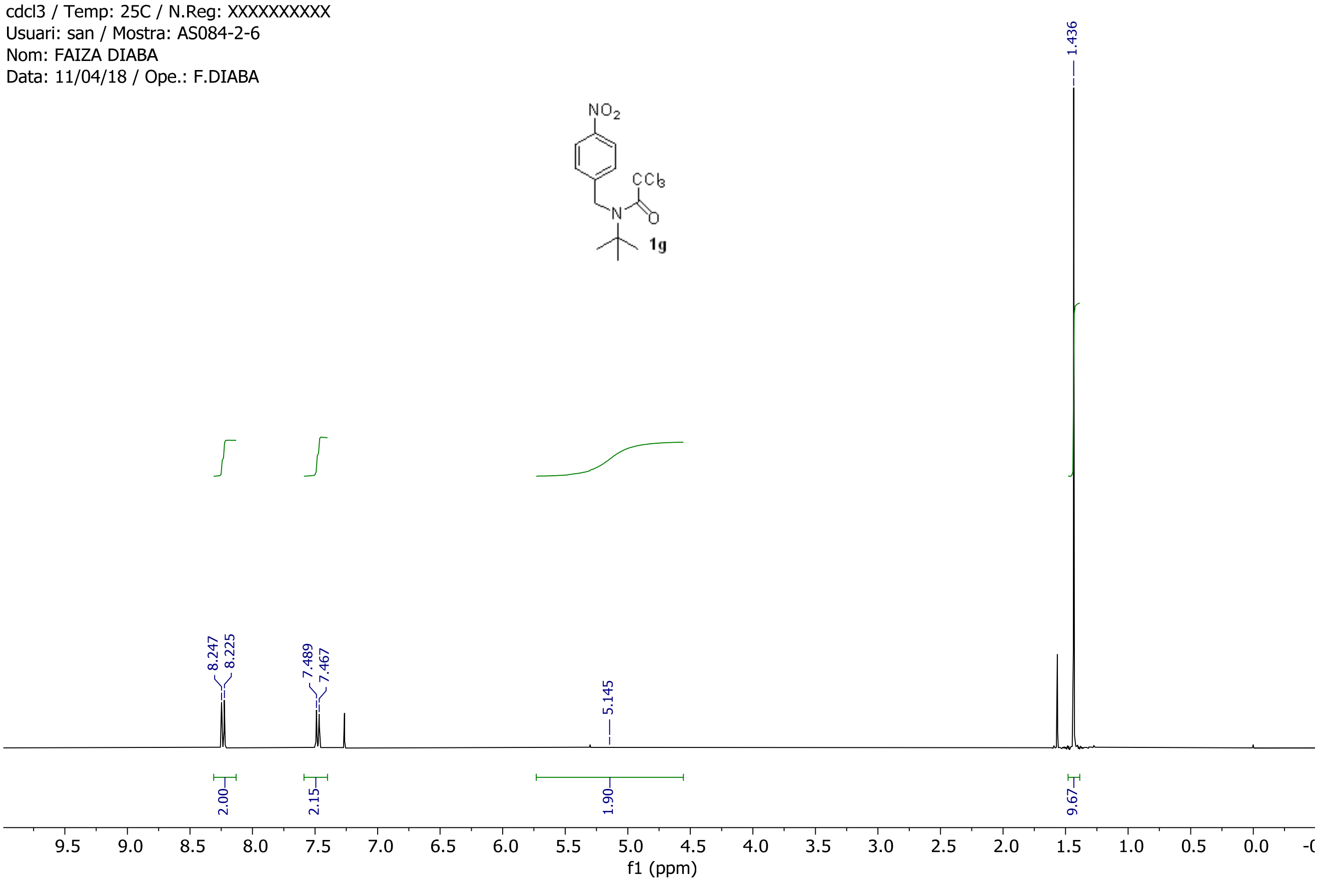

VNMRS400A_11042018_AS084-2-6-C13
VNMRS400F / Num.Inv. 205984
cdcl3 / Temp: 25C / N.Reg: XXXXXXXXXX
Usuari: san / Mostra: AS084-2-6
Nom: FAIZA DIABA
Data: 11/04/18 / Ope.: F.DIABA

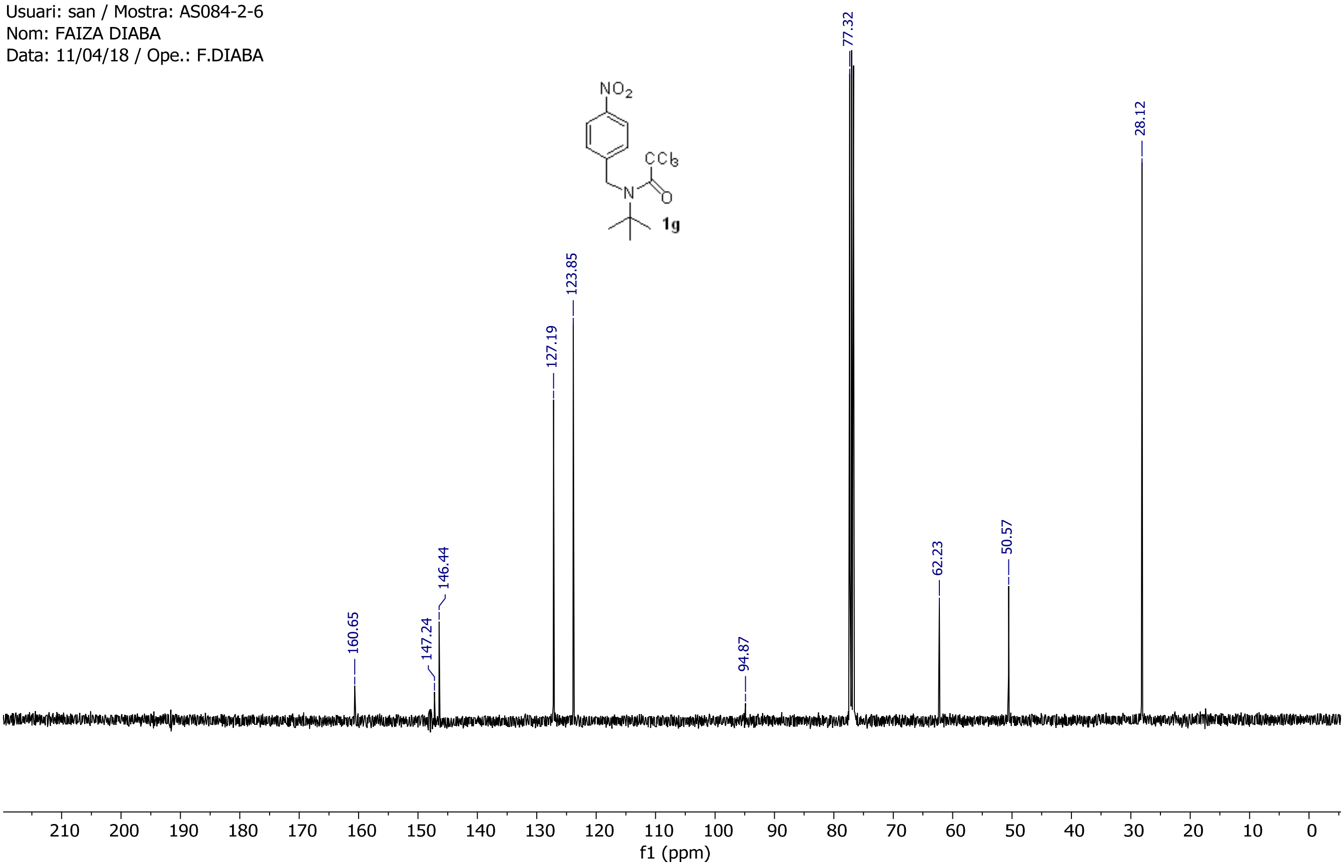

Figure S3

M400AFF_22052017_AS020-10-H1
M400F / Num.Inv. 1009191
cdcl3 / Temp: 25C / N.Reg: XXXXXXXXXX
Usuari: san / Mostra: AS020-10
Nom: FAIZA DIABA
Data: 22/05/17 / Ope.: F.DIABA

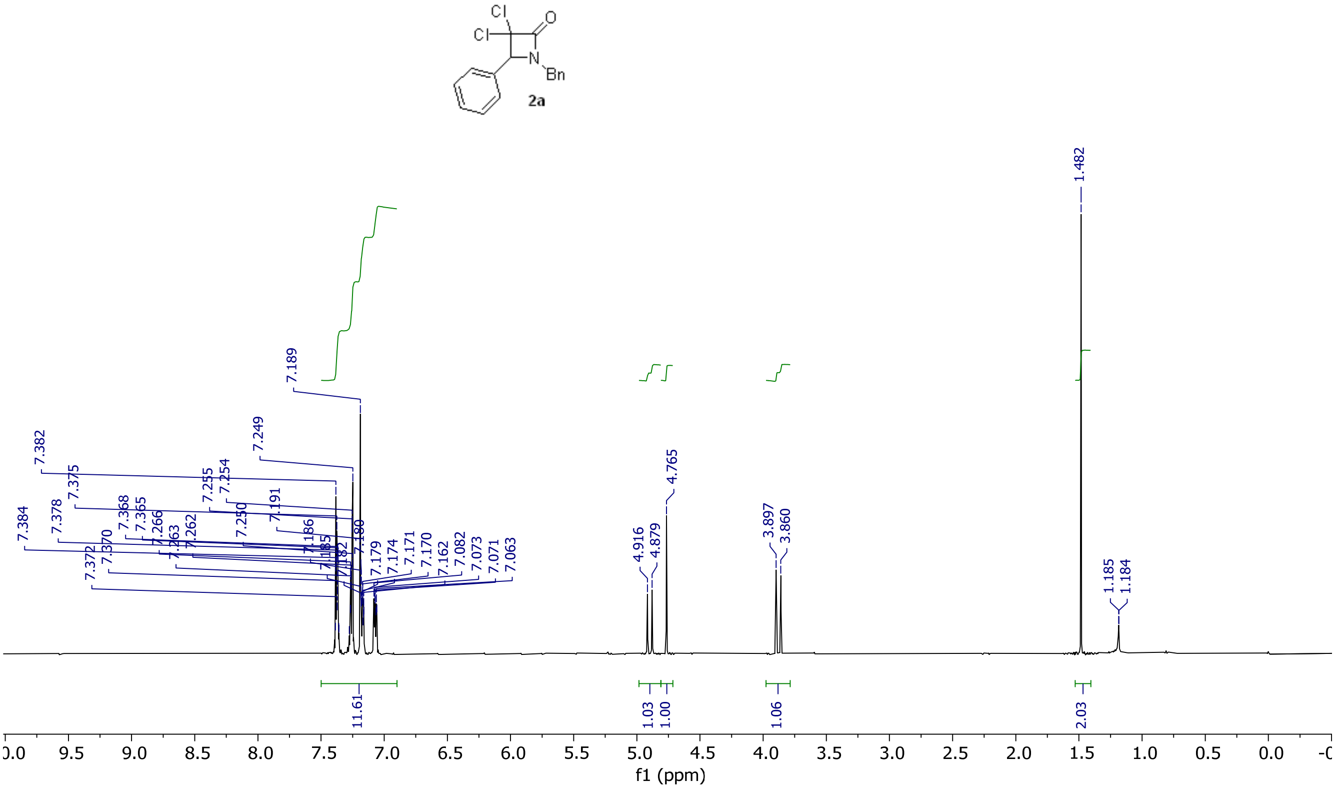

VNMRS400A_31052017_AC020-10-C13
VNMRS400F / Num.Inv. 205984
cdcl3 / Temp: 25C / N.Reg: XXXXXXXXXX
Usuari: san / Mostra: AC020-10
Nom: FAIZA DIABA
Data: 31/05/17 / Ope.: F.DIABA

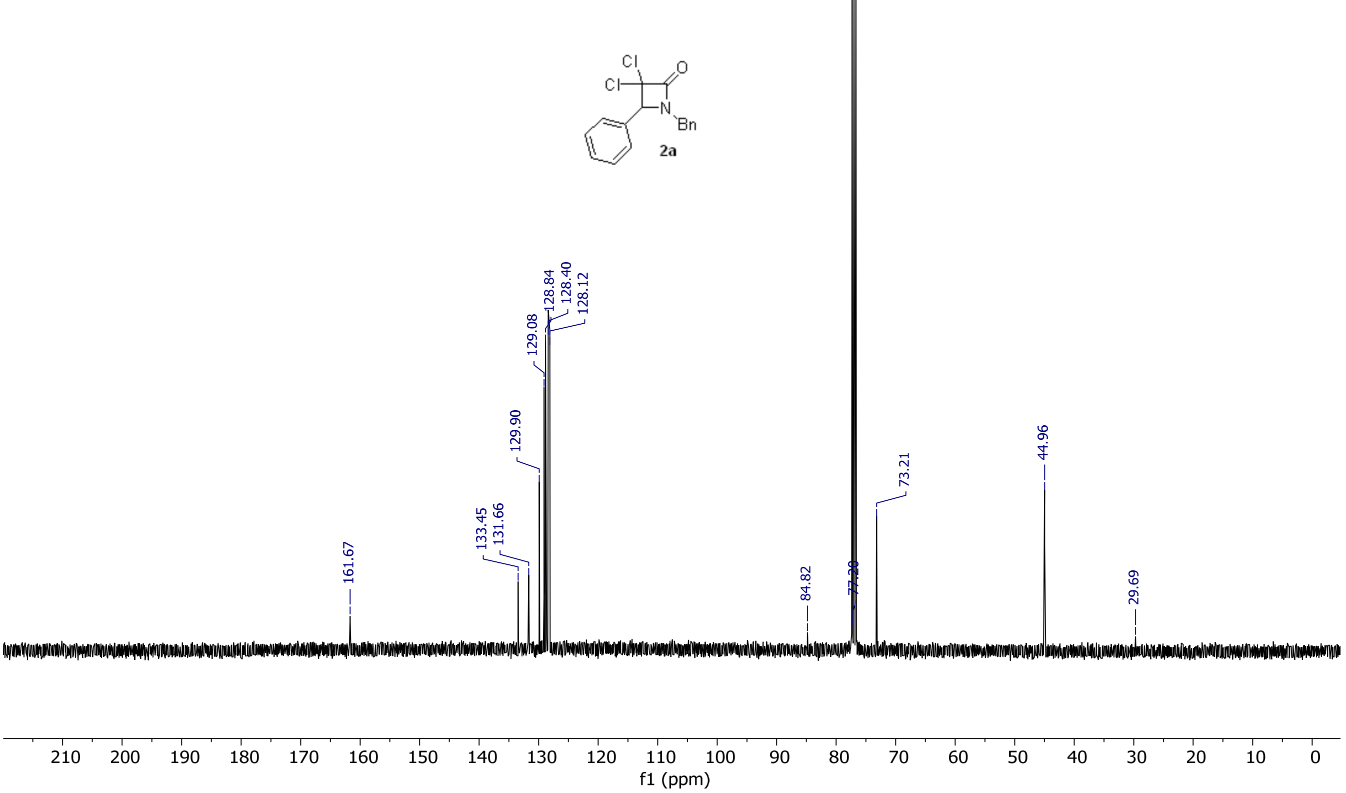

Figure S4

M400AFF_24072017_AS044-24-H1
M400F / Num.Inv. 1009191
CDCl3 / Temp: 25C / N.Reg: XXXXXXXXXX
Usuari: san / Mostra: AS044-24
Nom: FAIZA DIABA
Data: 24/07/17 / Ope.: F.DIABA

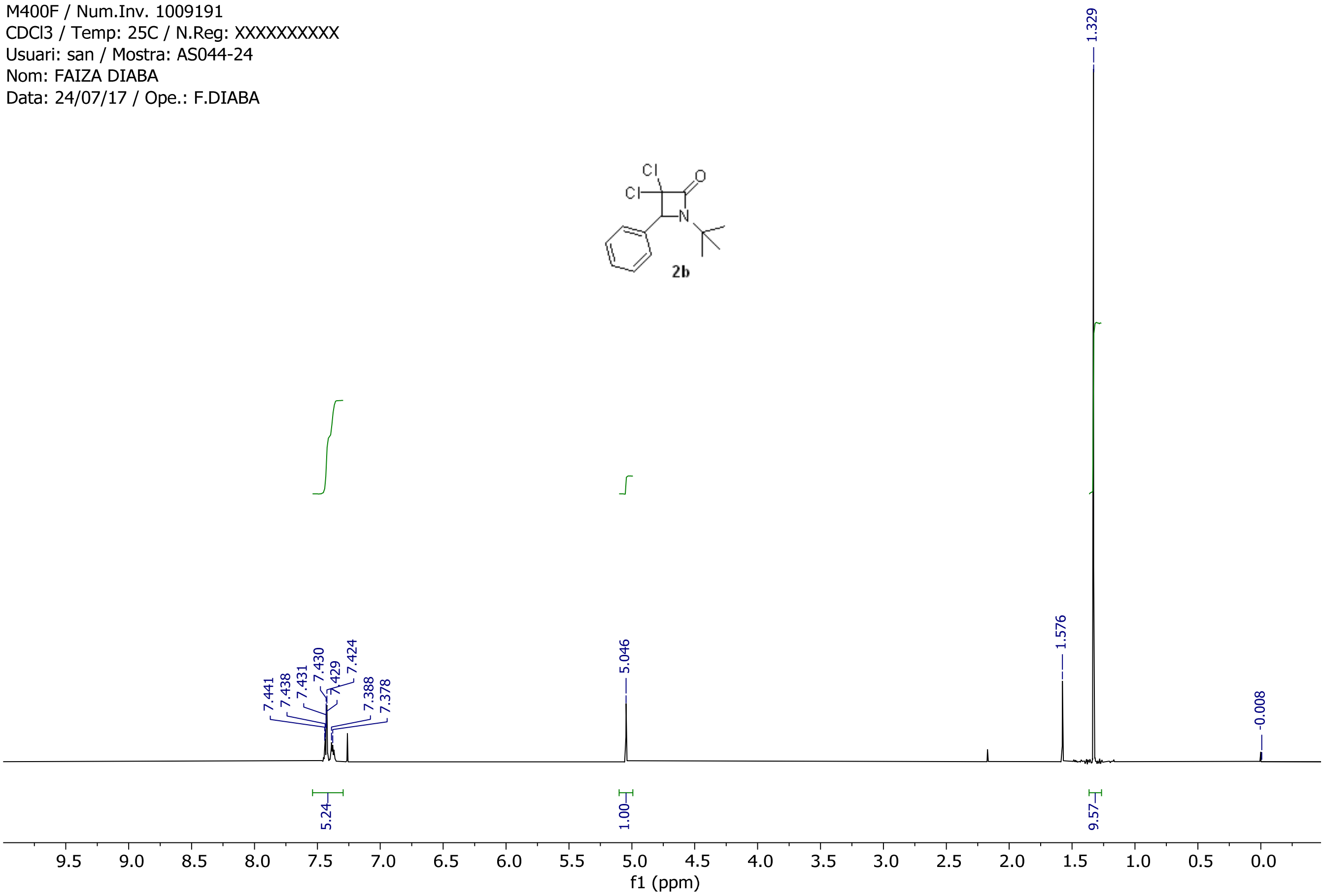

VNMRS400A_27072017_AS044-24-C13
VNMRS400F / Num.Inv. 205984
cdcl3 / Temp: 25C / N.Reg: XXXXXXXXXX
Usuari: san / Mostra: AS044-24
Nom: FAIZA DIABA
Data: 27/07/17 / Ope.: F.DIABA

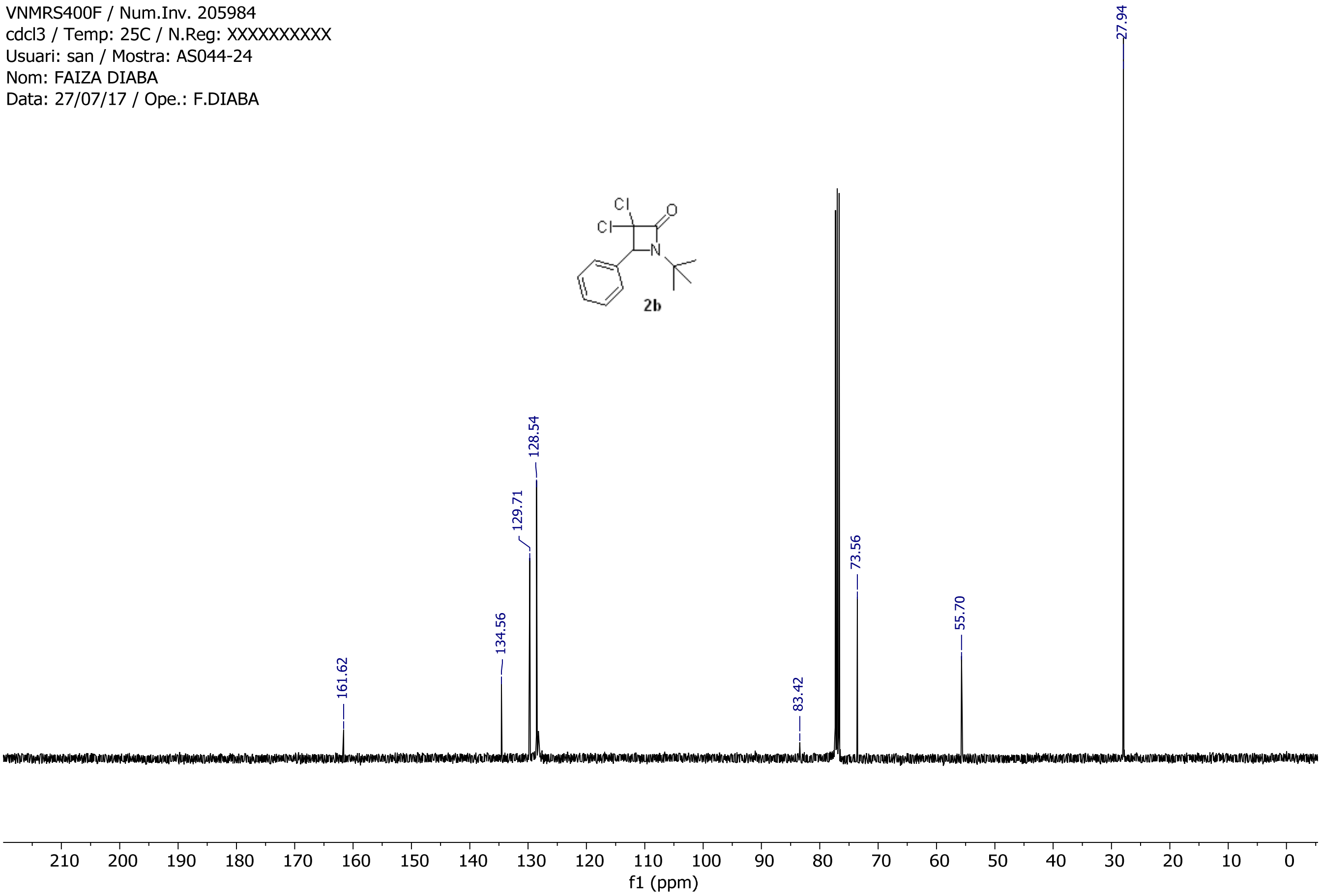

Figure S5

VNMRS400A_13012018_AS071-2-6-H1
VNMRS400F / Num.Inv. 205984
cdcl3 / Temp: 25C / N.Reg: XXXXXXXXXX
Usuari: san / Mostra: AS071-2-6
Nom: FAIZA DIABA
Data: 13/01/18 / Ope.: F.DIABA

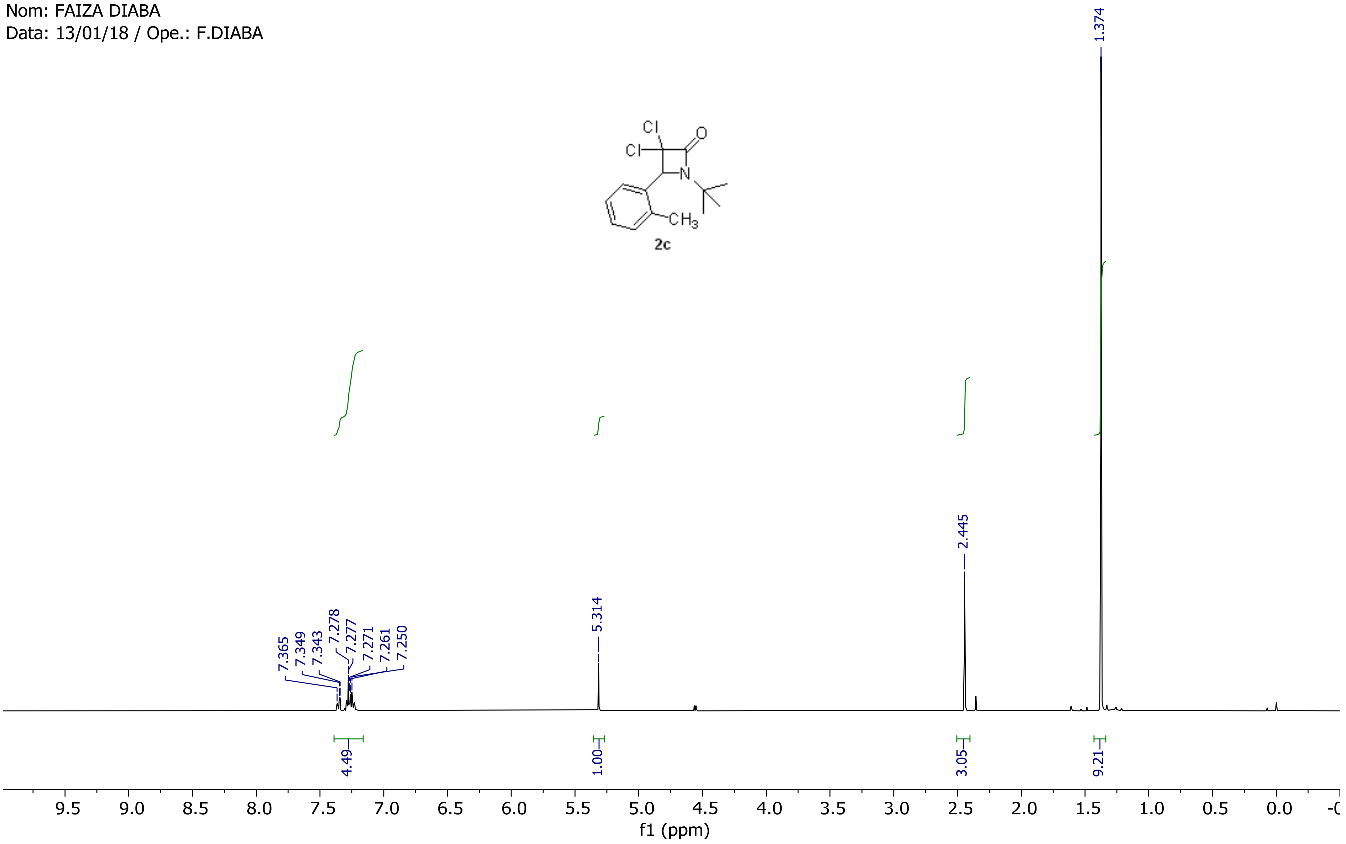

VNMRS400A_13012018_AS071-2-6-C13
VNMRS400F / Num.Inv. 205984
cdcl3 / Temp: 25C / N.Reg: XXXXXXXXXX
Usuari: san / Mostra: AS071-2-6
Nom: FAIZA DIABA
Data: 13/01/18 / Ope.: F.DIABA

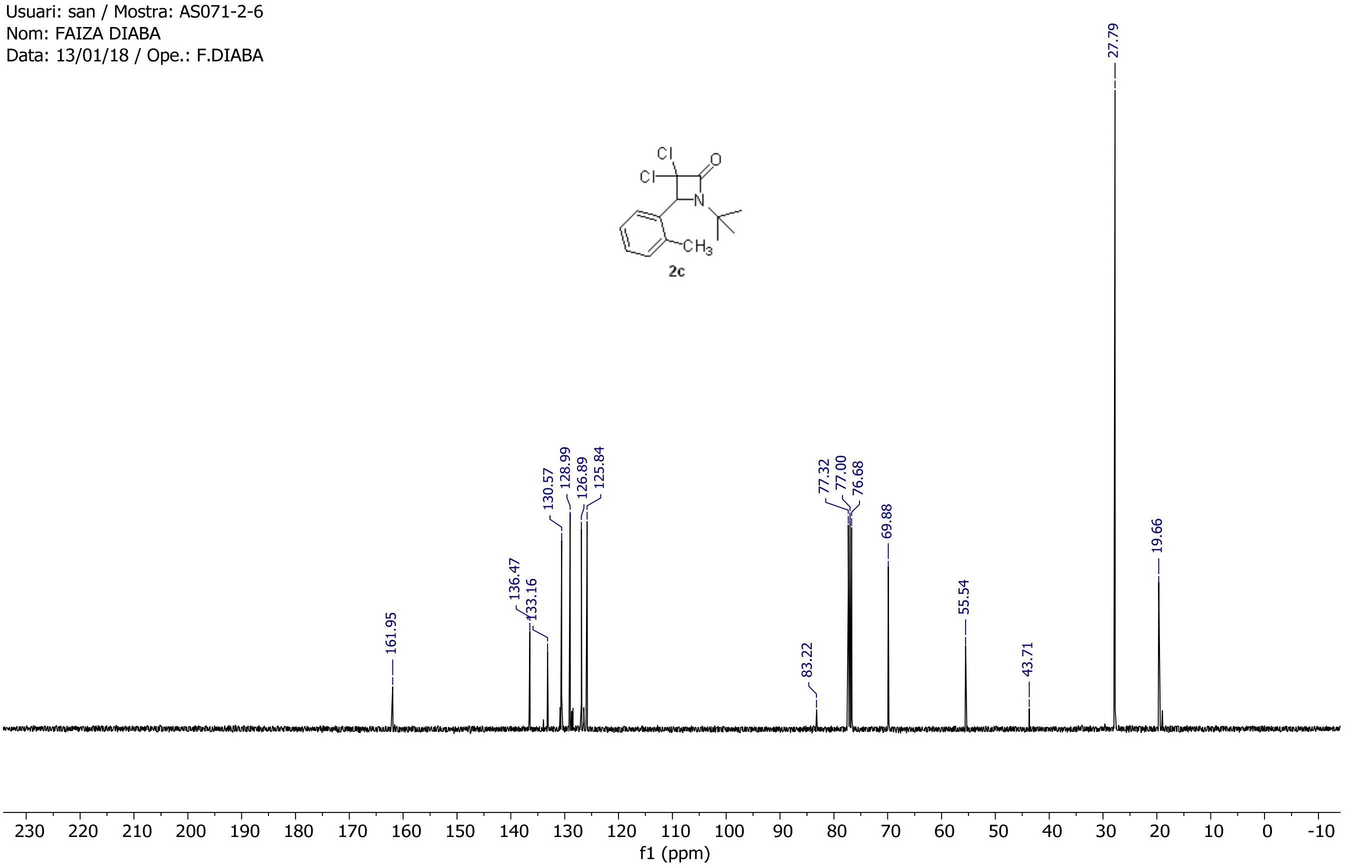

Figure S6

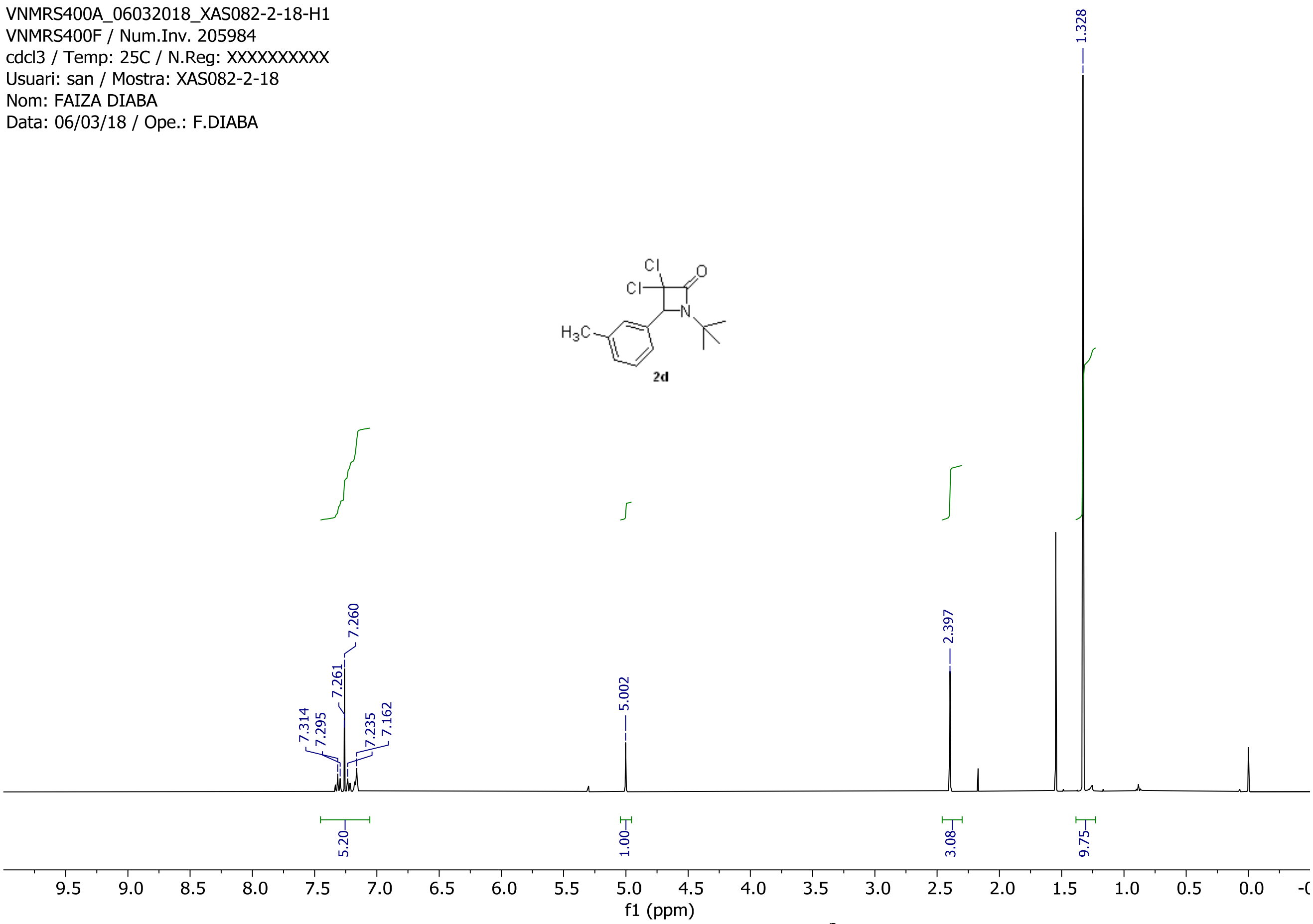
VNMRS400A_06032018_XAS082-2-18-H1
VNMRS400F / Num.Inv. 205984
cdcl3 / Temp: 25C / N.Reg: XXXXXXXXXX
Usuari: san / Mostra: XAS082-2-18
Nom: FAIZA DIABA
Data: 06/03/18 / Ope.: F.DIABA
2d
7.314
7.295
7.261
7.260
7.235
7.162
5.002
2.397
1.328
5.20
1.00
3.08
9.75
f1 (ppm)

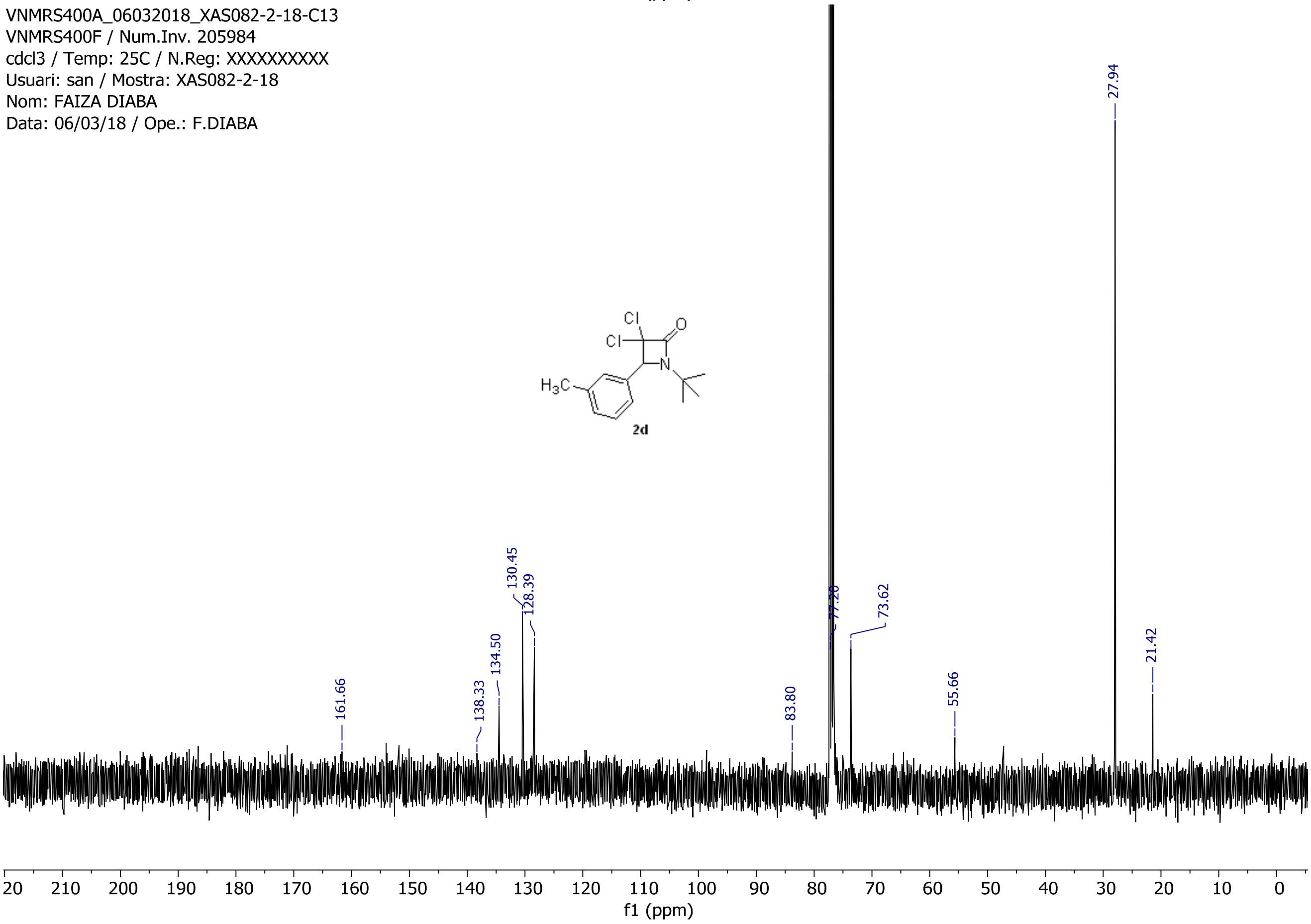
VNMRS400A_06032018_XAS082-2-18-C13
VNMRS400F / Num.Inv. 205984
cdcl3 / Temp: 25C / N.Reg: XXXXXXXXXX
Usuari: san / Mostra: XAS082-2-18
Nom: FAIZA DIABA
Data: 06/03/18 / Ope.: F.DIABA
2d
161.66
138.33
134.50
130.45
128.39
83.80
73.62
55.66
27.94
21.42
f1 (ppm)

Figure S7

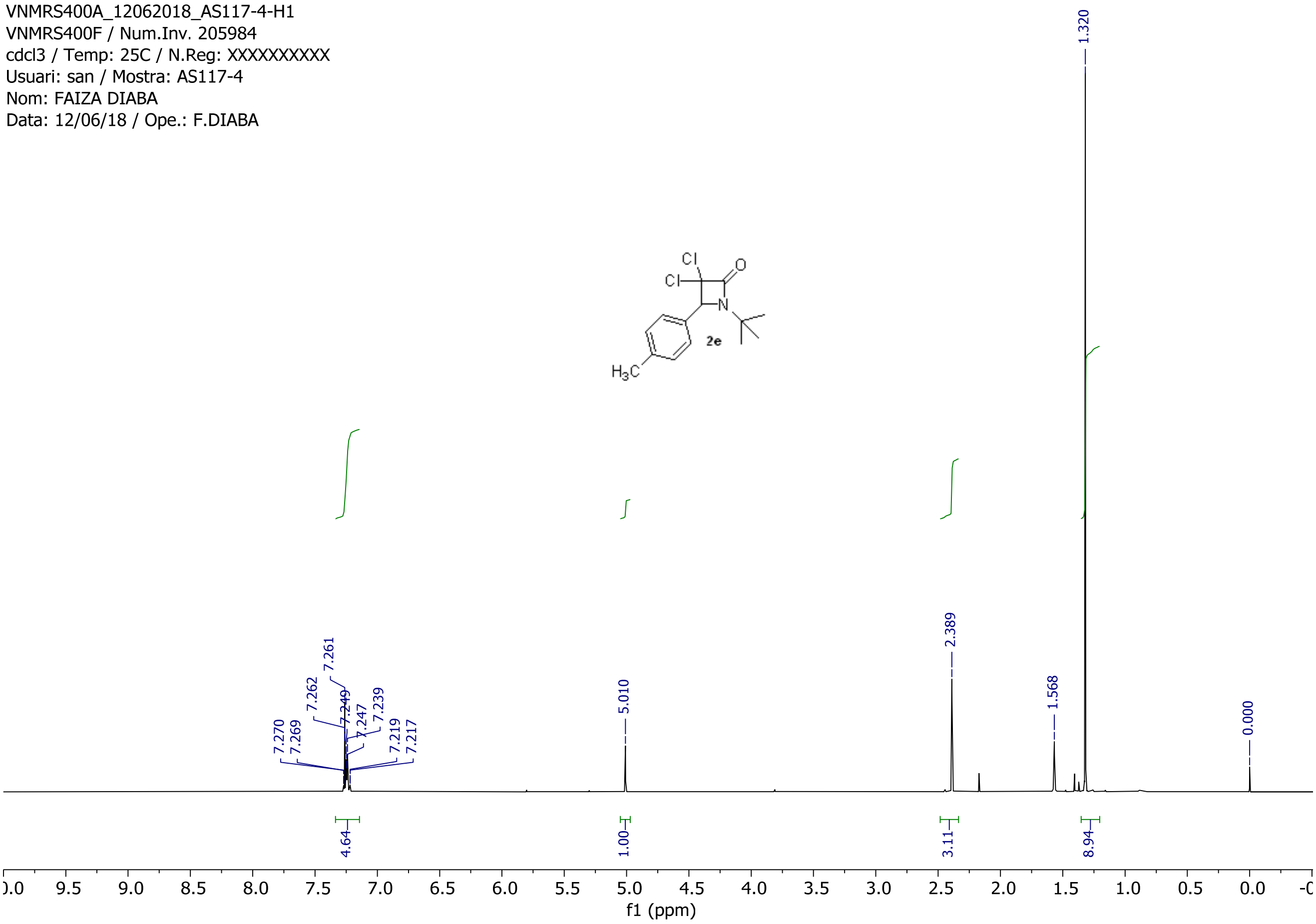
VNMRS400A_12062018_AS117-4-H1
VNMRS400F / Num.Inv. 205984
cdcl3 / Temp: 25C / N.Reg: XXXXXXXXXX
Usuari: san / Mostra: AS117-4
Nom: FAIZA DIABA
Data: 12/06/18 / Ope.: F.DIABA
Cl
Cl
O
N
2e
H_3C
7.270
7.269
7.262
7.261
7.249
7.247
7.239
7.219
7.217
5.010
2.389
1.568
1.320
0.000
4.64
1.00
3.11
8.94
f1 (ppm)

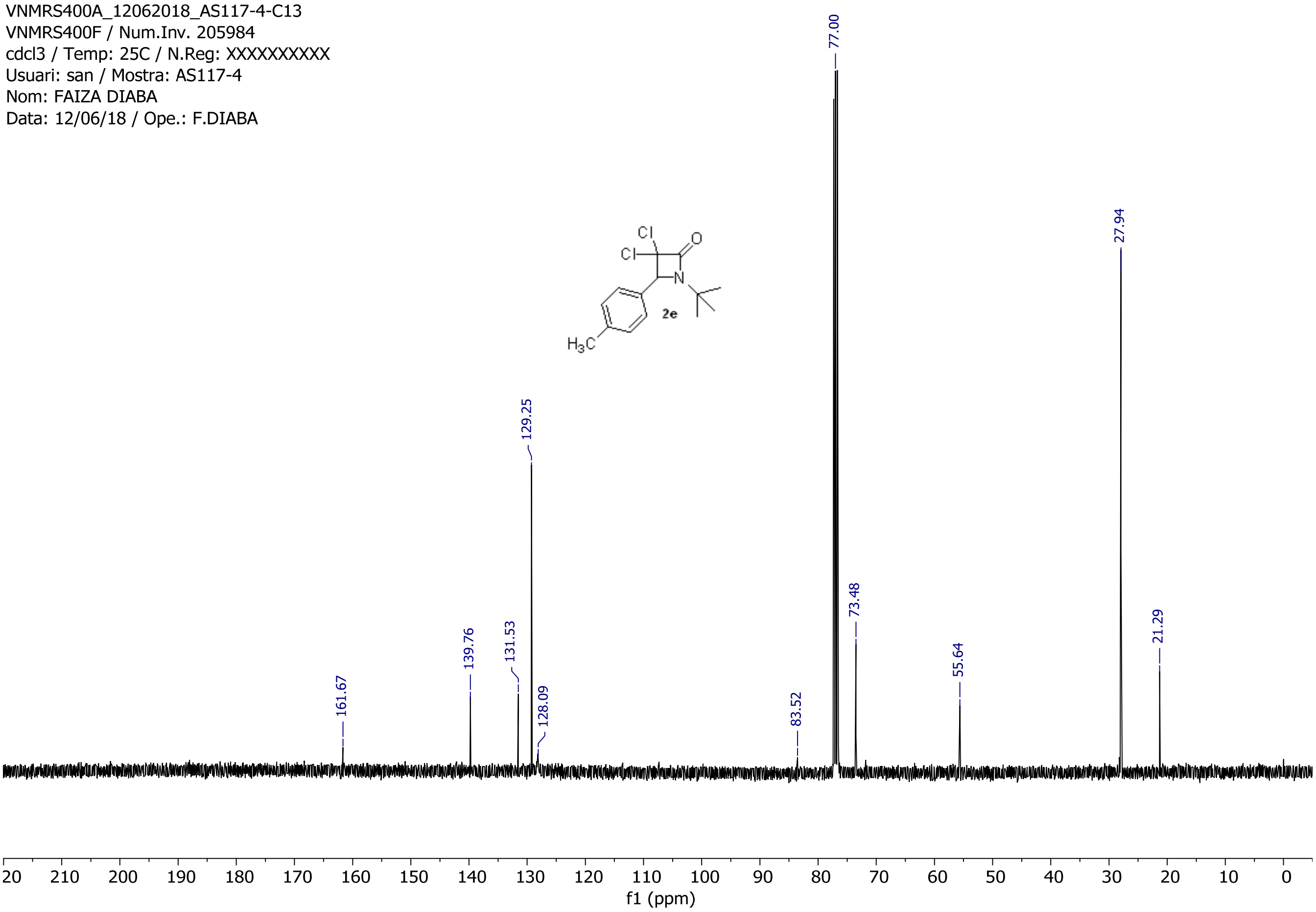
VNMRS400A_12062018_AS117-4-C13
VNMRS400F / Num.Inv. 205984
cdcl3 / Temp: 25C / N.Reg: XXXXXXXXXX
Usuari: san / Mostra: AS117-4
Nom: FAIZA DIABA
Data: 12/06/18 / Ope.: F.DIABA
Cl
Cl
O
N
2e
H_3C
161.67
139.76
131.53
129.25
128.09
83.52
77.00
73.48
55.64
27.94
21.29
f1 (ppm)

Figure S8

VNMRS400A_28112017_AS068-19-H1
VNMRS400F / Num.Inv. 205984
cdcl3 / Temp: 25C / N.Reg: XXXXXXXXXX
Usuari: san / Mostra: AS068-19
Nom: FAIZA DIABA
Data: 28/11/17 / Ope.: F.DIABA

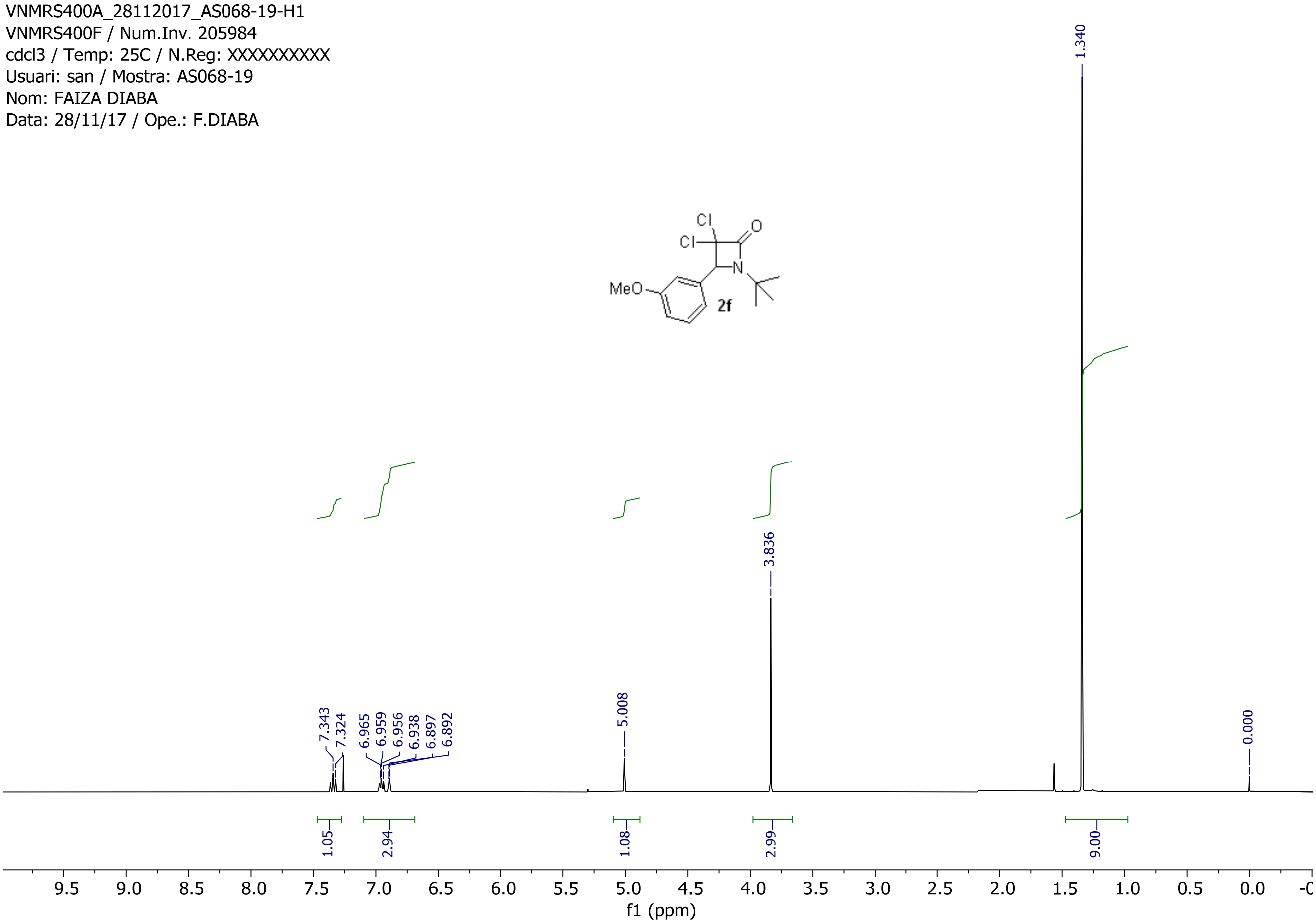

VNMRS400A_28112017_AS068-19-C13
VNMRS400F / Num.Inv. 205984
cdcl3 / Temp: 25C / N.Reg: XXXXXXXXXX
Usuari: san / Mostra: AS068-19
Nom: FAIZA DIABA
Data: 28/11/17 / Ope.: F.DIABA

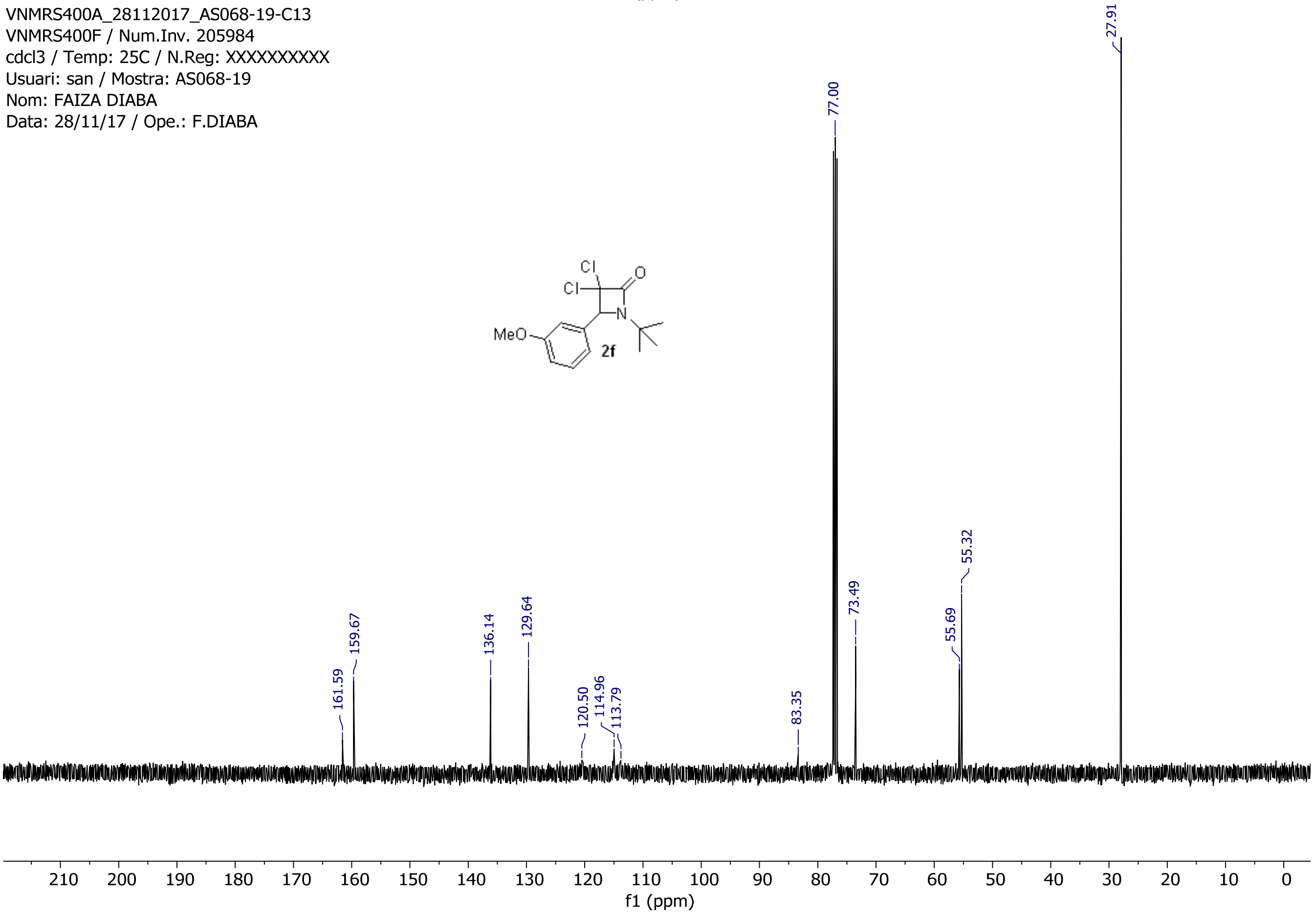

Figure S9

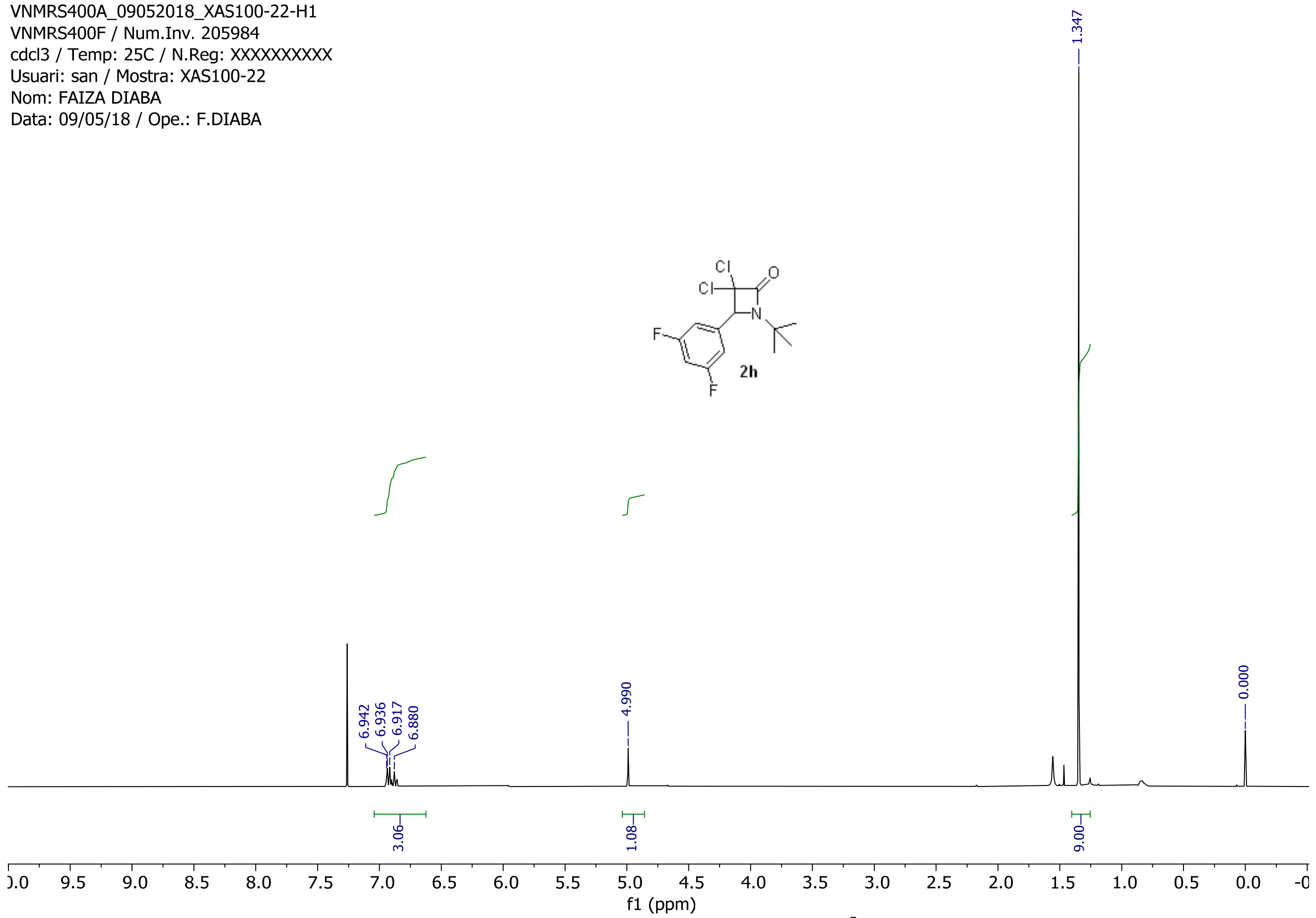
VNMRS400A_09052018_XAS100-22-H1
VNMRS400F / Num.Inv. 205984
cdcl3 / Temp: 25C / N.Reg: XXXXXXXXXX
Usuari: san / Mostra: XAS100-22
Nom: FAIZA DIABA
Data: 09/05/18 / Ope.: F.DIABA
Cl
Cl
O
N
F
F
2h
6.942
6.936
6.917
6.880
4.990
1.347
0.000
3.06
1.08
9.00
f1 (ppm)

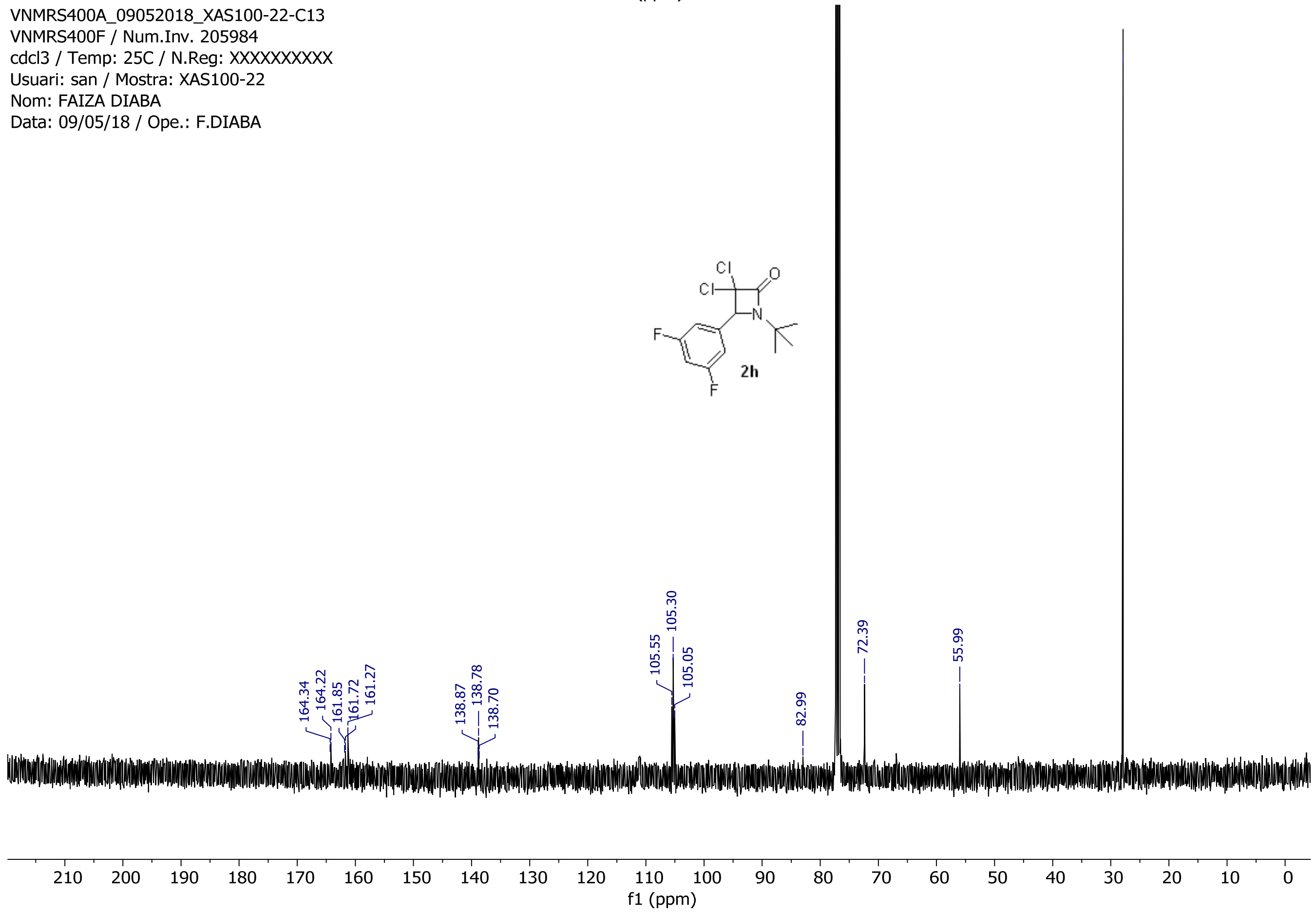
VNMRS400A_09052018_XAS100-22-C13
VNMRS400F / Num.Inv. 205984
cdcl3 / Temp: 25C / N.Reg: XXXXXXXXXX
Usuari: san / Mostra: XAS100-22
Nom: FAIZA DIABA
Data: 09/05/18 / Ope.: F.DIABA
Cl
Cl
O
N
F
F
2h
164.34
164.22
161.85
161.72
161.27
138.87
138.78
138.70
105.55
105.30
105.05
82.99
72.39
55.99
f1 (ppm)

Figure S10

VNMRS400A_04122018_as156-13-H1
VNMRS400F / Num.Inv. 205984
cdcl3 / Temp: 25C / N.Reg: XXXXXXXXXX
Usuari: san / Mostra: as156-13
Nom: FAIZA DIABA
Data: 04/12/18 / Ope.: F.DIABA

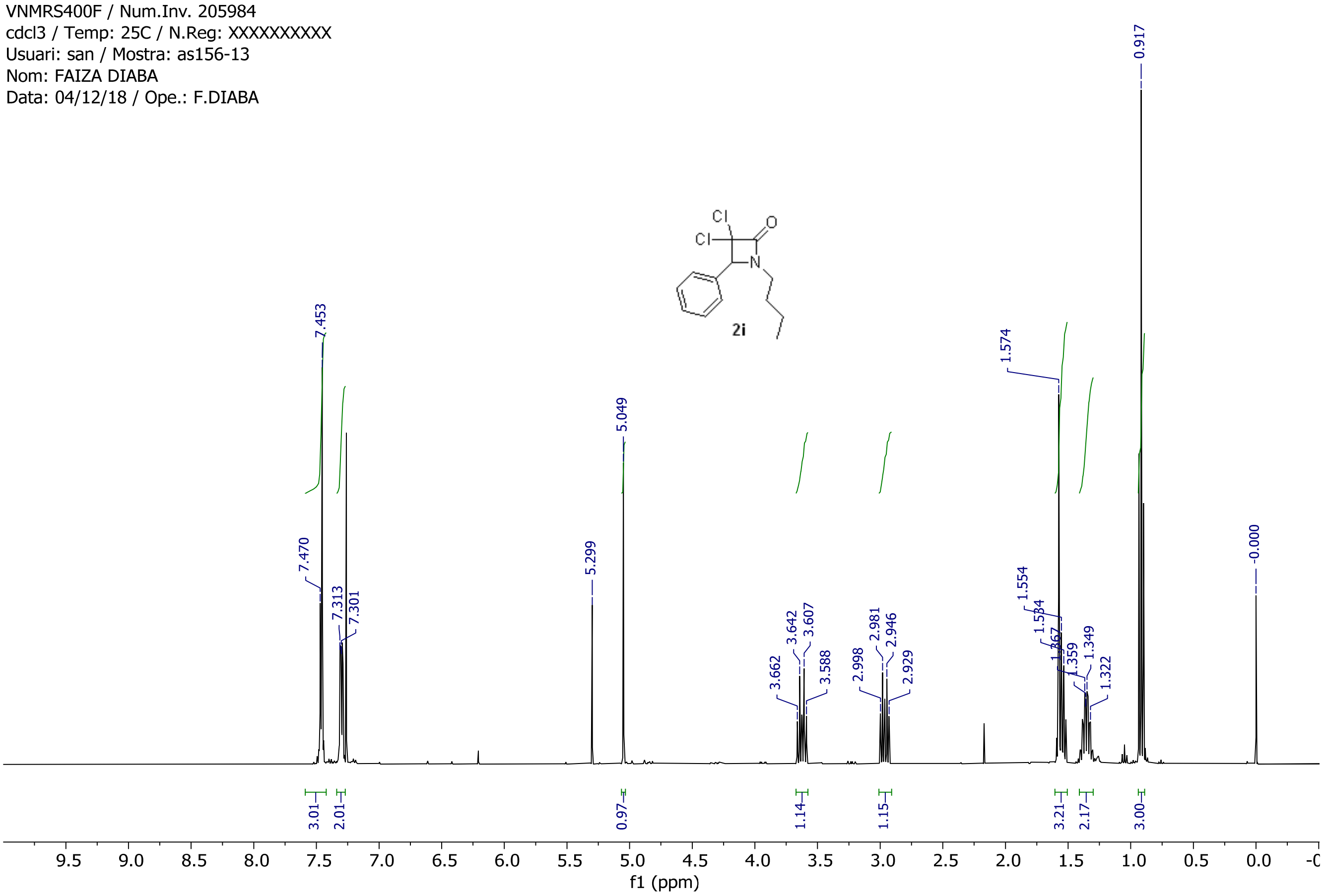

VNMRS400A_04122018_as156-13-C13
VNMRS400F / Num.Inv. 205984
cdcl3 / Temp: 25C / N.Reg: XXXXXXXXXX
Usuari: san / Mostra: as156-13
Nom: FAIZA DIABA
Data: 04/12/18 / Ope.: F.DIABA

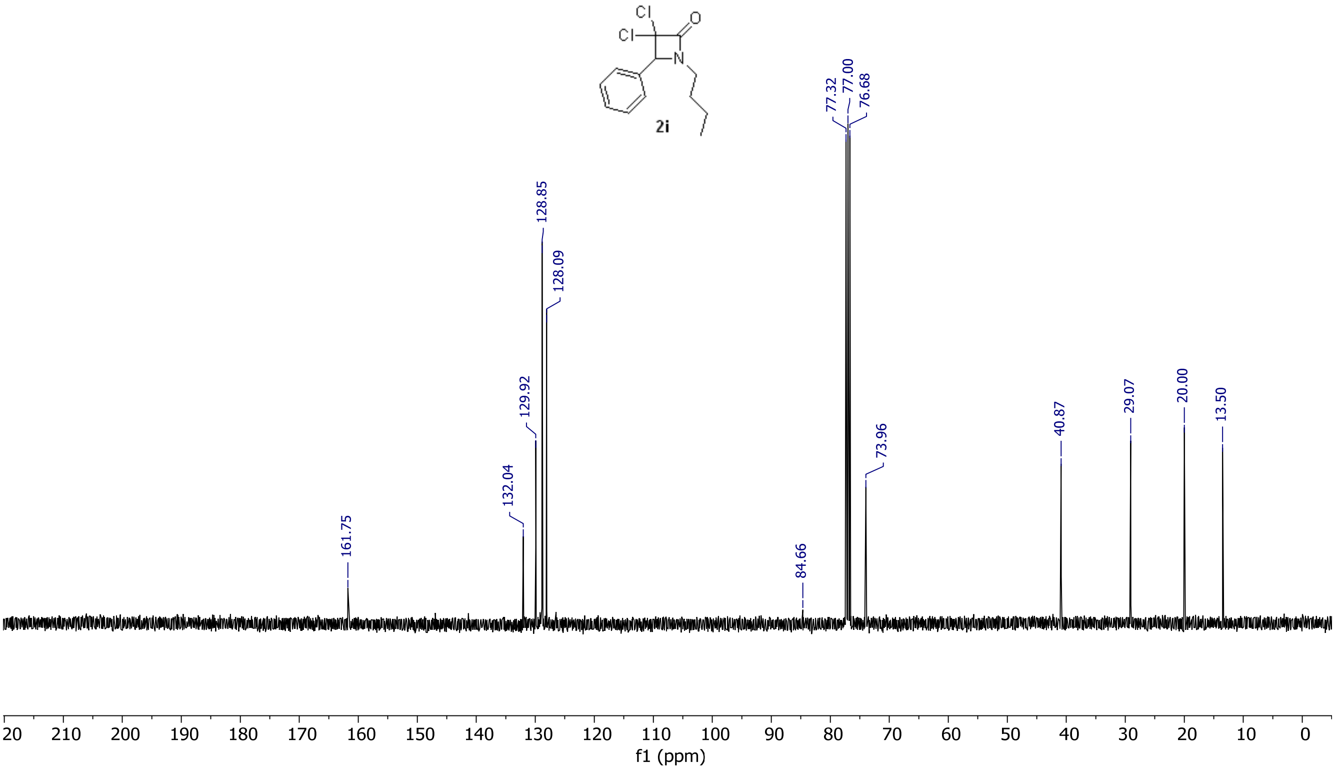

Figure S11

VNMRS400A_01122018_AS154-13-H1
VNMRS400F / Num.Inv. 205984
cdcl3 / Temp: 25C / N.Reg: XXXXXXXXXX
Usuari: san / Mostra: AS154-13
Nom: FAIZA DIABA
Data: 01/12/18 / Ope.: F.DIABA

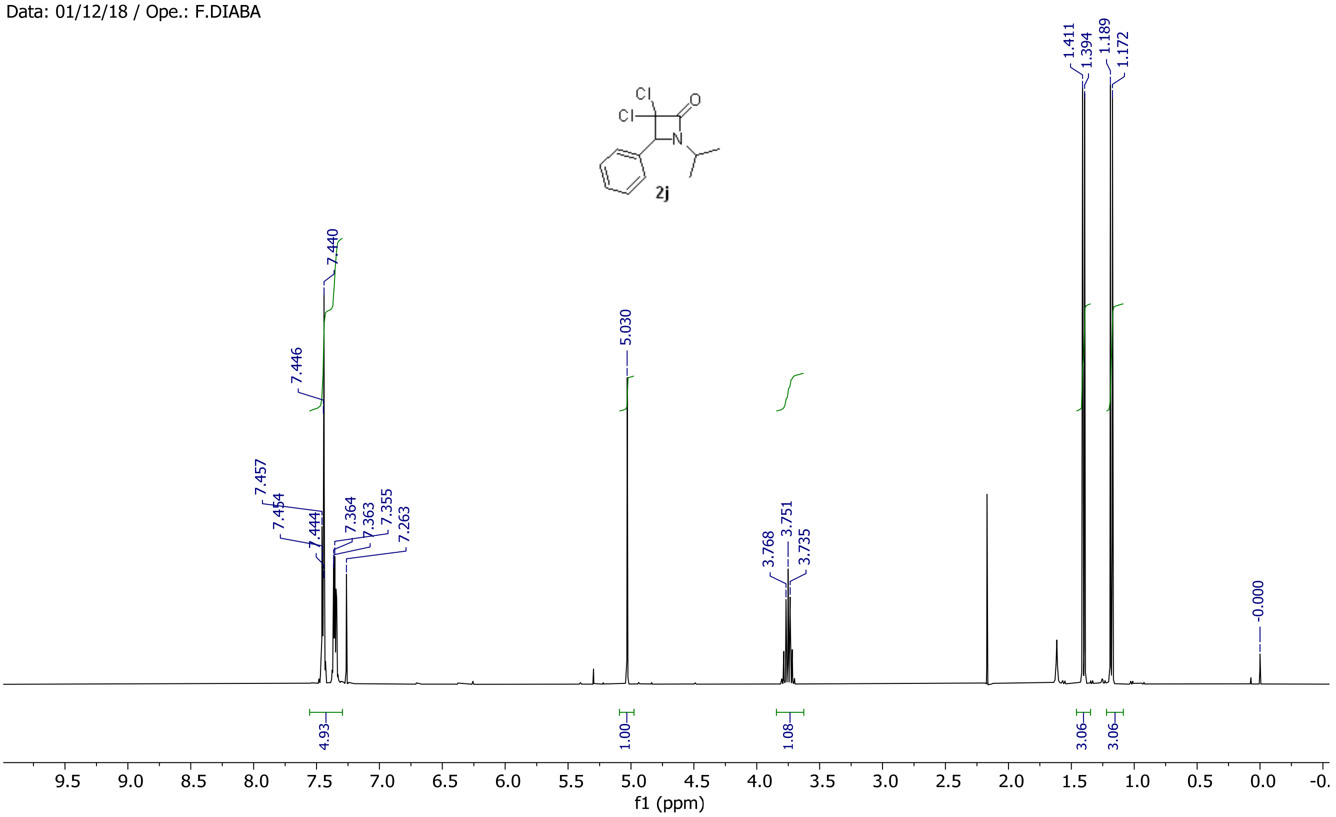

VNMRS400A_01122018_AS154-13-C13
VNMRS400F / Num.Inv. 205984
cdcl3 / Temp: 25C / N.Reg: XXXXXXXXXX
Usuari: san / Mostra: AS154-13
Nom: FAIZA DIABA
Data: 01/12/18 / Ope.: F.DIABA

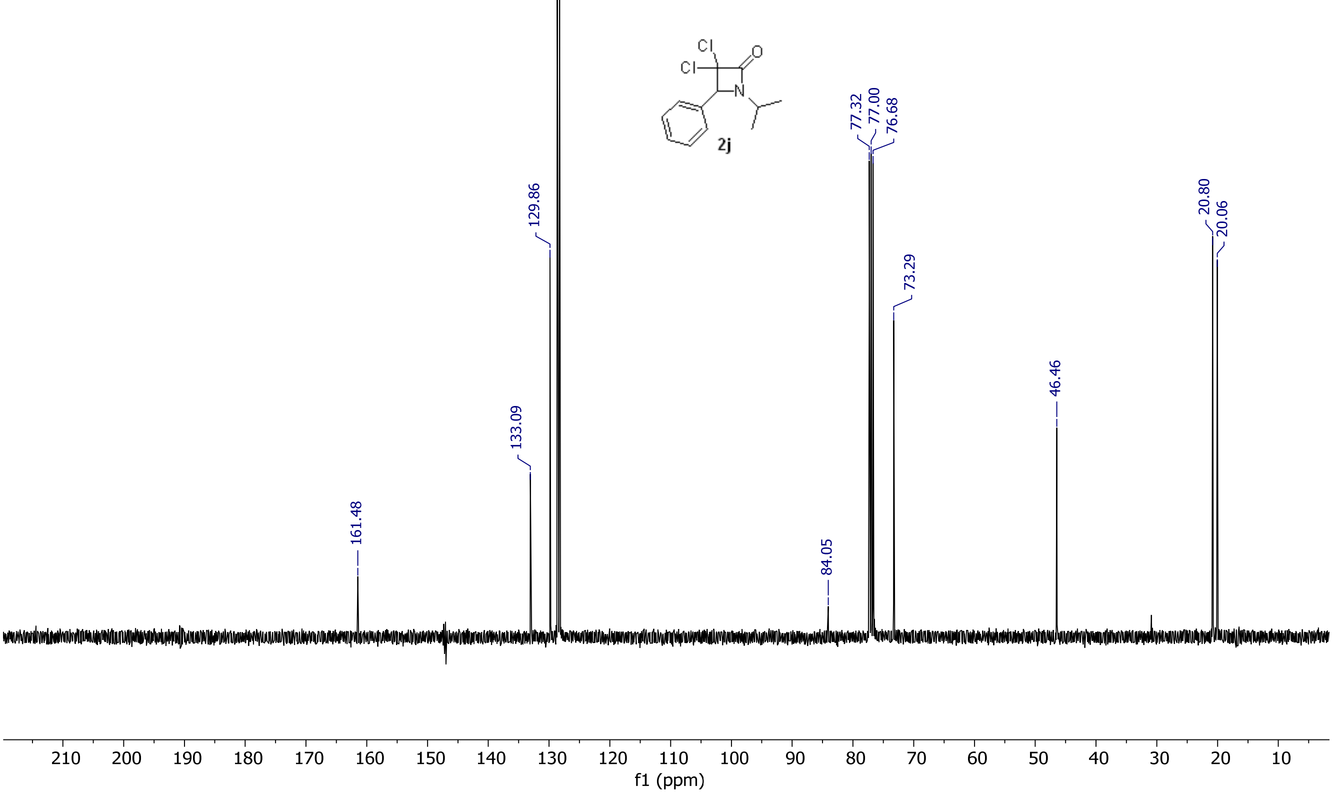

Figure S12

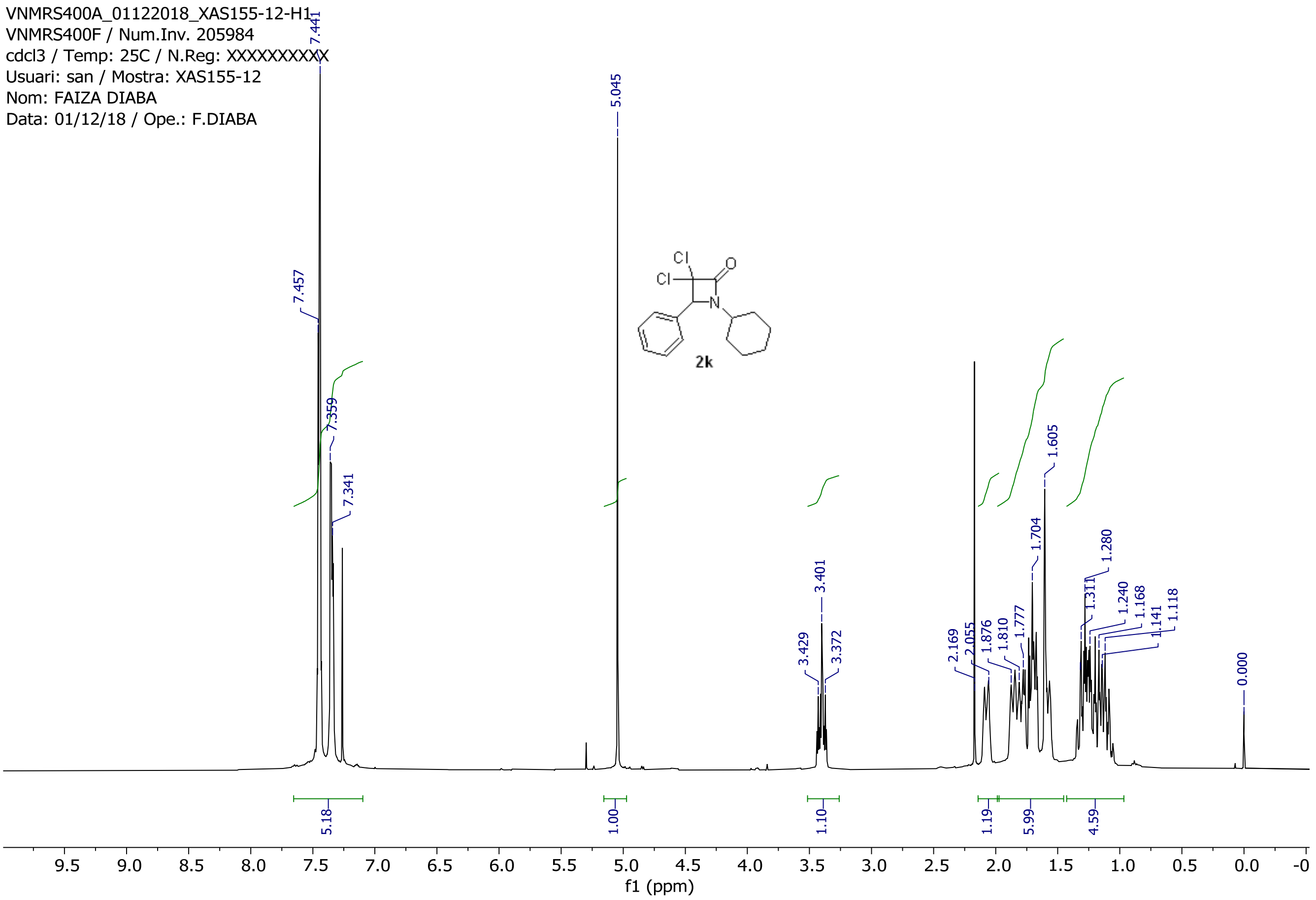
VNMRS400A_01122018_XAS155-12-H1
VNMRS400F / Num.Inv. 205984
cdcl3 / Temp: 25C / N.Reg: XXXXXXXXXX
Usuari: san / Mostra: XAS155-12
Nom: FAIZA DIABA
Data: 01/12/18 / Ope.: F.DIABA
7.457
7.441
7.359
7.341
5.045
3.429
3.401
3.372
2.169
2.055
1.876
1.810
1.777
1.704
1.605
1.311
1.280
1.240
1.168
1.141
1.118
0.000
Cl
Cl
O
N
2k
5.18
1.00
1.10
1.19
5.99
4.59
f1 (ppm)

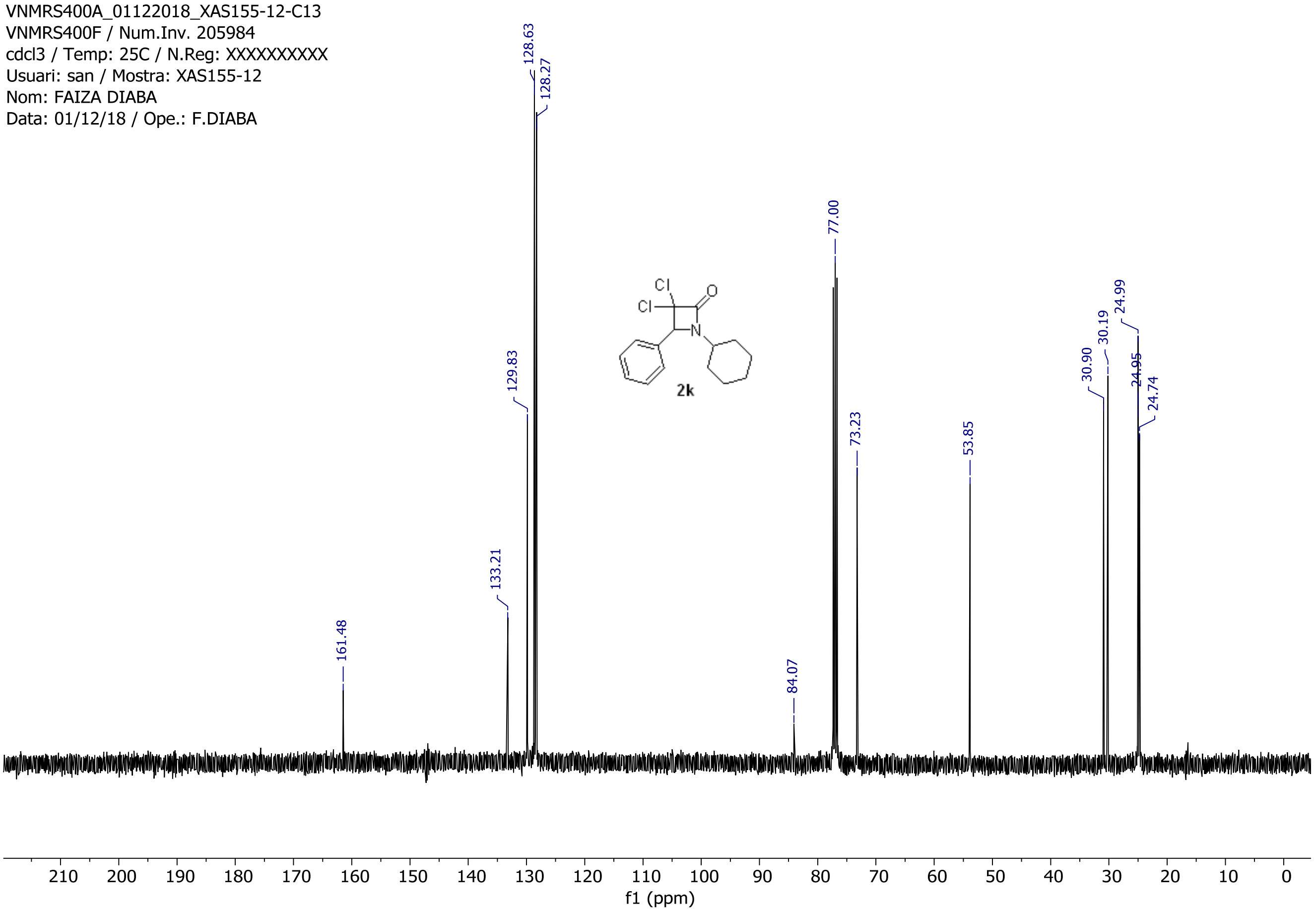
VNMRS400A_01122018_XAS155-12-C13
VNMRS400F / Num.Inv. 205984
cdcl3 / Temp: 25C / N.Reg: XXXXXXXXXX
Usuari: san / Mostra: XAS155-12
Nom: FAIZA DIABA
Data: 01/12/18 / Ope.: F.DIABA
161.48
133.21
129.83
128.63
128.27
84.07
77.00
73.23
53.85
30.90
30.19
24.99
24.95
24.74
Cl
Cl
O
N
2k
f1 (ppm)

Figure S13
